# Evolution of high-level resistance during low-level antibiotic exposure

**DOI:** 10.1038/s41467-018-04059-1

**Published:** 2018-04-23

**Authors:** Erik Wistrand-Yuen, Michael Knopp, Karin Hjort, Sanna Koskiniemi, Otto G. Berg, Dan I. Andersson

**Affiliations:** 1 Department of Medical Biochemistry and Microbiology, Uppsala University, 75237 Uppsala, Sweden; 2 Department of Cell and Molecular Biology, Uppsala University, 75237 Uppsala, Sweden

## Abstract

It has become increasingly clear that low levels of antibiotics present in many environments can select for resistant bacteria, yet the evolutionary pathways for resistance development during exposure to low amounts of antibiotics remain poorly defined. Here we show that *Salmonella enterica* exposed to sub-MIC levels of streptomycin evolved high-level resistance via novel mechanisms that are different from those observed during lethal selections. During lethal selection only *rpsL* mutations are found, whereas at sub-MIC selection resistance is generated by several small-effect resistance mutations that combined confer high-level resistance via three different mechanisms: (i) alteration of the ribosomal RNA target (*gidB* mutations), (ii) reduction in aminoglycoside uptake (*cyoB*,* nuoG*, and *trkH* mutations), and (iii) induction of the aminoglycoside-modifying enzyme AadA (*znuA* mutations). These results demonstrate how the strength of the selective pressure influences evolutionary trajectories and that even weak selective pressures can cause evolution of high-level resistance.

## Abstract (web summary)

Mutational antibiotic resistance can emerge under either high or low antibiotic levels. Here, the authors show several small-effect resistance mutations are combined to confer high-level resistance in *Salmonella enterica* exposed to sub-MIC levels of streptomycin.

## Introduction

Irrespective of whether antibiotics are used to treat infections in humans or animals, for growth promotion in animals, aquaculture, or plant production, a substantial fraction of these antibiotics will ultimately end up in the environment^1^. Thus, there are many environments such as wastewater, sludge, soil, and river water where bacteria are exposed for long periods of time to low concentrations of polluting antibiotics that are present because of anthropogenic influences^2–7^. Furthermore, low antibiotic concentrations (below the minimal inhibitory concentration, MIC) might be present in certain human/animal body compartments and tissues during therapeutic or growth promotion use. Previous studies showed that low levels of antibiotics (sub-MIC) can enrich for pre-existing resistant mutants in a bacterial population, indicating that certain antibiotics, disinfectants, and heavy metals could contribute to resistance evolution at concentrations that are several hundred-fold below the MIC^8–13^.

While many studies have examined the genetics of mutational antibiotic resistance selected at high levels (>MIC) of antibiotics, less is known about the effects of long-term exposure to low levels (<MIC) of antibiotics^14–19^. When susceptible bacteria are exposed to antibiotic concentrations above the MIC they will die or stop growing, and only bacteria where resistance mutations were present prior to antibiotic exposure will be able to grow. In contrast, at sub-MIC concentrations of antibiotics the bacteria can still grow while they are under selection, generating a potentially different trajectory of evolution with progressive increase in resistance through the step-wise accumulation of resistance mutations with individually smaller effects. During selection at high concentrations of streptomycin the most common resistance mutations are target alteration mutations in the gene *rpsL*, encoding ribosomal protein S12^20,21^, that confer high-level resistance >1024 mg L^−1^^22,23^. Mutations in the electron transport chain that confer a small colony variant (SCV) phenotype can also cause a moderate increase in resistance (16–96 mg L^−1^), which is often linked to a severe reduction in growth rate^24–26^. In this work, we investigated the evolution and genetics of antibiotic resistance selected during long-term exposure to streptomycin at sub-MIC level of the drug in *Salmonella enterica*, an important bacterial pathogen and model system for studies of antibiotic resistance. Results show that resistance evolution below the MIC of streptomycin occurs via different mechanisms compared to lethal selection and that high-level resistance can evolve also when bacteria are exposed to low antibiotic concentrations (sub-MIC).

## Results

### Mutant selection above MIC

Many previous studies have shown that *rpsL* mutants are the major type of mutants found at selection above the MIC^22,^^23^. We reconfirmed these results and showed that when 10 independent cultures of susceptible *Salmonella enterica* serovar Typhimurium LT2 strain (designated *S*. *typhimurium* throughout the text) were selected on Mueller–Hinton (MH) agar for streptomycin resistance at 200 mg L^−1^ of streptomycin (50× above the MIC), 10/10 mutants had mutations in *rpsL* (amino acid substitutions: six K42R, one K42N, one K42T, and two K87R) that conferred the resistance. Whole-genome sequencing of six independent isolates confirmed that mutants selected on high streptomycin concentrations on agar plates had only *rpsL* mutations. Furthermore, we also performed a serial passage experiment (100 generations) in liquid MH containing 200 mg L^−1^ streptomycin. Whole-genome sequencing of five populations showed that the only resistance conferring mutations present in them were *rpsL* mutations (K42R). Thus, for 11 independent selections at high streptomycin levels only *rpsL* mutants were selected.

### Mutant selection below MIC

To study evolution of antibiotic resistance in a susceptible bacterial population below MIC, 20 independent lineages of the streptomycin susceptible wild-type *S*. *typhimurium* were serially passaged for 900 generations in MH medium containing 1 mg L^−1^ of streptomycin, corresponding to 1/4 of the MIC of the susceptible wild type. The concentration of streptomycin used causes an approximately 3% reduction in competitive growth rate of the susceptible wild type and was chosen to provide a weak sub-MIC selection. This estimation was based on previous work^8^, where in a serial passage competition experiment 1 mg L^−1^ streptomycin balances the 3% fitness cost conferred by an *rpsL* (K42R) mutation. Serial passage occurred every 24 h by transfer of 1 µl of overnight culture (5 × 10^9^ cells/ml) to 1 ml of culture medium, generating a bottleneck of 5 × 10^6^ cells during transfer. After serial passage, bacteria were plated on MH agar plates with different concentrations of streptomycin (8, 16, 32, 64, 96, 128, 192, and 256 mg L^−1^) to estimate the frequency of cells with different resistance levels. The populations were heterogeneous with regard to resistance and several of the lineages contained subpopulations (approximately 0.1−1% of the cells) with high levels of resistance (MIC of streptomycin >96 mg L^−1^). Clones with increased resistance were single-colony isolated from six independent lineages, and these purified clones were further analyzed. The MICs of streptomycin varied between 64 mg L^−1^ and >1024 mg L^−1^ depending on the mutant. Four clones (obtained from four different lineages) with the highest streptomycin resistances (>192 mg L^−1^) were investigated further. In control experiments, where bacteria were serially passaged for 1000 generations in the absence of streptomycin, no streptomycin-resistant mutants were found^27^.

### The spectra of mutations at sub-MIC and >MIC selection differ

To identify the mutations responsible for the increased resistance to streptomycin, the four clones with the highest resistance levels were whole-genome sequenced, and their sequences were compared to that of the ancestral strain to identify potential point mutations and structural rearrangements (deletions, duplications, insertions, and inversions). The results showed that all four strains had mutator phenotypes, with roughly 100 different point mutations per genome (Supplementary Table 1). The mutator phenotypes are likely to be caused by mutations in the DNA mismatch repair system since the strains had mutations in the *mutSLH* genes^28^.

To identify which mutations contributed to the high-level streptomycin resistance, we first examined if there were mutations present in the serially passaged strains that have previously been implicated in resistance. As shown by our present data and previous studies^22,23^, during selection for streptomycin resistance at high levels of streptomycin, mainly mutations in the gene *rpsL*, encoding the 30S ribosomal subunit protein S12, are found. However, none of the clones isolated here from serial passage at sub-MIC concentrations had *rpsL* mutations. Instead, all of them had loss-of-function mutations in the *gidB* gene, encoding a 16S rRNA methyltransferase that has been linked to low levels of streptomycin resistance in *Salmonella* and *Mycobacterium tuberculosis*^25,29^. In addition, the strain with the highest streptomycin resistance also carried a mutation in the gene *trkH* encoding a potassium symporter, a gene previously shown to be involved in aminoglycoside resistance in *Escherichia coli*^15,30^.

To elucidate which additional mutations might be involved in the high-level resistance, we identified genes that were mutated in more than one lineage with the assumption that repeated occurrence of mutations in a specific gene is likely to reflect a response to the antibiotic selection, rather than fixation of a random non-selected mutation (however, it should be noted that media adaptation mutations could also show repeated appearance in independent lineages, see below). Thus, besides the *gidB* and *trkH* mutations the strains with the highest streptomycin resistances (Table 1 and Supplementary Table 1) had also accumulated mutations in genes involved in the respiratory electron transport chain, such as *nuoG* and *nuoE*, encoding subunits of NADH dehydrogenase and *cyoB*, encoding the cytochrome o oxidase subunit I. Two of the strains also carried mutations in the *znuAB* genes encoding proteins involved in zinc uptake. This generated a list of five candidate mutations: *gidB*, *trkH*, *nuoG*, *cyoB*, and *znuA* that were examined further.

**Table 1:** Isolated resistant mutants

Strain	Genotype	Streptomycin MIC (mg L^−1^)	Reference
DA6192	Wild type (parent)	4	Strain collection
DA23868^a^	Mutant isolated by plating serial passage population on 256 mg L^−1^ streptomycin and picking a single colony (for complete genotype, see Supplementary Table 1)	512	This study
DA23869^a^	Mutant isolated by plating serial passage population on 256 mg L^−1^ streptomycin and picking a single colony (for complete genotype, see Supplementary Table 1)	>1024	This study
DA23872^a^	Mutant isolated by plating serial passage population on 192 mg L^−1^ streptomycin and picking a single colony (for complete genotype, see Supplementary Table 1)	512	This study
DA23874^a^	Mutant isolated by plating serial passage population on 128 mg L^−1^ streptomycin and picking a single colony (for complete genotype, see Supplementary Table 1)	192	This study
DA23877^a^	Mutant isolated by plating serial passage population on 96 mg L^−1^ streptomycin and picking a single colony (for complete genotype, see Supplementary Table 1)	96	This study
DA23879^a^	Mutant isolated by plating serial passage population on 96 mg L^−1^ streptomycin and picking a single colony (for complete genotype, see Supplementary Table 1)	64	This study
DA25308	DA23868, *aadA::cat*	4	This study
DA25310	DA23869, *aadA::cat*	16	This study
DA25312	DA23872, *aadA::cat*	8	This study
DA25314	DA23874, *aadA::cat*	8	This study
DA26068	DA23868, *relA::*Tn*10d*Tet	512	This study
DA26070	DA23869, *relA::*Tn*10d*Tet	384	This study
DA26072	DA23872, *relA::*Tn*10d*Tet	128	This study
DA26074	DA23874, *relA::*Tn*10d*Tet	128	This study
DA27220	DA23868, *relA::*Tn*10d*Tet, *∆spoT::cat*	192	This study
DA27222	DA23869, *relA::* Tn*10d*Tet, *∆spoT::cat*	256	This study
DA27224	DA23872, *relA::*Tn*10d*Tet, *∆spoT::cat*	96	This study
DA27226	DA23874, *relA::*Tn*10d*Tet, *∆spoT::cat*	48	This study

Streptomycin resistance of *S. typhimurium* clones isolated from serial passage and the effects of *aadA* and *relA*, *spoT* inactivation mutations on resistance. MIC values presented are the median values of 2–5 Etests from biological replicates

^a^ Note that the four whole-genome sequenced clones contained many mutations (see Supplementary Table 1 for list of all mutations)

### Combination of five mutations generated high-level resistance

**Fig. 1:**
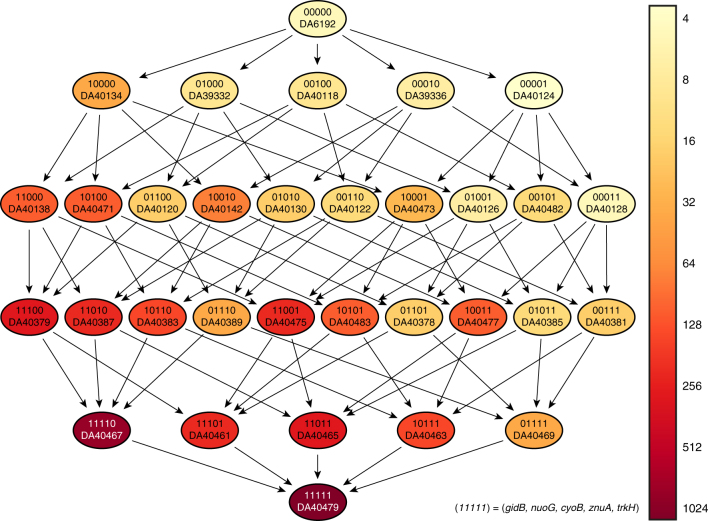
The fitness landscape of streptomycin resistance. The color scale indicates the MIC of streptomycin in mg L^−1^ for each reconstructed mutant. The genotype of each strain is written as a five-bit binary code, where 0 and 1 represent the wild-type and mutant sequences, respectively. See Table 2 for complete strain genotypes

**Fig. 2:**
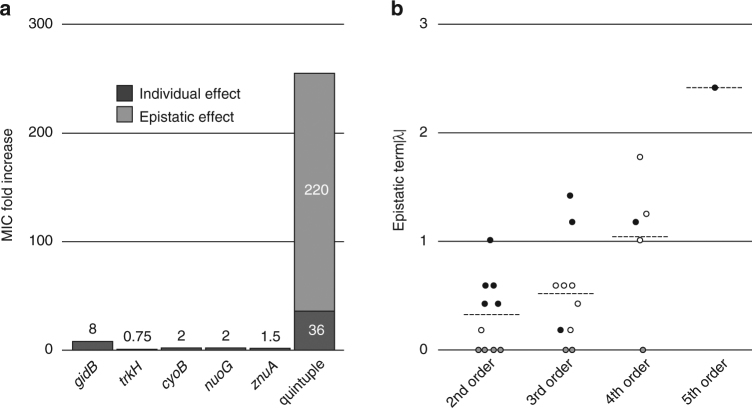
Epistatic interactions. **a** Five mutations generate higher resistance than expected from their individual effects. The MIC increase based on individual additive effects is indicated in dark gray, while fold increase based on epistatic interactions is indicated in light gray. **b** The epistatic interactions increase with higher orders. Positive epistasis (filled circles) predominates at the 2nd and 5th order, while negative epistasis (empty circles) predominates at 3rd and 4th order. The strongest epistasis is observed in the quintuple mutant. Gray circles represent no epistatic interactions. The full dataset of epistatic interactions can be found in Supplementary Table 2

The MIC of streptomycin for the *S*. *typhimurium* strain (DA6192) used as a starting ancestral strain for the sub-MIC cycling was 4 mg L^−1^ and the isolated resistant mutants had MIC values ranging from 192 to >1024 mg L^−1^ (Table 1). To examine how the putative resistance mutations contributed to the highest level of resistance (>1024 mg L^−1^), we reconstructed all possible combinations of the five candidate genes: *gidB*, *cyoB*, *nuoG*, *trkH*, and *znuA* (identified as described above), generating 32 different mutants (Fig. 1, Table 2). Of the five reconstructed single mutants only the mutation in the gene *gidB* conferred a significant increase in resistance to streptomycin. Thus, the loss-of-function mutation *gidB* (Q169*) (where * represents a stop codon) gave a MIC of 32 mg L^−1^ for streptomycin, similar to previously reported values^25^. In contrast, the other single mutants gave no (*trkH*), 1.5-fold (*znuA*), or 2-fold (*cyoB*, *nuoG*) increases in resistance (Table 2 and Fig. 1). By combining several of the mutations generating all potential double, triple, quadruple, and quintuple mutants, a step-wise increase in resistance was observed, reaching up to >1024 mg L^−1^. Thus, adding mutations in the respiratory chain to a *gidB* (Q169*) genetic background increased the resistance further, where mutations in *cyoB* (G283D) or *nuoG* (D264G) individually increased the MIC to 96 mg L^−1^, while the combination of both gave a MIC of 256 mg L^−1^ (Table 2 and Fig. 1). Reconstruction of the selected *znuA* (S19fs) (where “fs” represents a frame shift) mutation in a genetic background of *gidB* (Q169*), *cyoB* (G283D), and *nuoG* (D264G) increased the MIC even further to 768 mg L^−1^ (Table 2 and Fig. 1). Finally, the addition of the *trkH* (T99A) mutation increased the resistance even further to a MIC of >1024 mg L^−1^. A comparison of the resistance levels of the single mutants with the higher-order mutants showed that there were strong epistatic interactions between the mutations to generate the high-level resistance (Fig. 2 and Supplementary Table 2). Thus, the expected additive resistance (without epistasis) is a 36-fold increase in MIC in the quintuple mutant compared to the wild type, while the observed increase was >220-fold (Fig. 2a).

**Table 2:** Reconstructed strains

Strain	*gidB*	*trkH*	*cyoB*	*nuoG*	*znuA*	Streptomycin MIC (mg L^−1^)	Streptomycin MIC with ∆*aadA*	Streptomycin MIC with *relA::Tn10 spoT::cat*
DA6192	wt	wt	wt	wt	wt	4	2	1.5
DA40134	Q169*					32	4	16
DA39332				D264G		8	3	6
DA40118			G283D			8	4	8
DA39336					S19fs	6	2	1.5
DA40124		T99A				3	2	2
DA40138	Q169*			D264G		96	8	48
DA40471	Q169*		G283D			96	12	48
DA40120			G283D	D264G		16	8	16
DA40142	Q169*				S19fs	64	4	24
DA40130				D264G	S19fs	16	4	8
DA40122			G283D		S19fs	12	6	6
DA40473	Q169*	T99A				24	6	48
DA40126		T99A		D264G		6	4	6
DA40482		T99A	G283D			12	6	12
DA40128		T99A			S19fs	4	2	3
DA40379	Q169*		G283D	D264G		256	16	96
DA40387	Q169*			D264G	S19fs	192	8	96
DA40383	Q169*		G283D		S19fs	128	8	96
DA40389			G283D	D264G	S19fs	32	12	16
DA40475	Q169*	T99A		D264G		192	12	64
DA40483	Q169*	T99A	G283D			96	16	48
DA40378		T99A	G283D	D264G		16	12	16
DA40477	Q169*	T99A			S19fs	96	4	24
DA40385		T99A		D264G	S19fs	12	4	12
DA40381		T99A	G283D		S19fs	16	6	12
DA40467	Q169*		G283D	D264G	S19fs	768	24	256
DA40461	Q169*	T99A	G283D	D264G		192	24	96
DA40465	Q169*	T99A		D264G	S19fs	256	8	96
DA40463	Q169*	T99A	G283D		S19fs	128	12	128
DA40469		T99A	G283D	D264G	S19fs	32	6	12
DA40479	Q169*	T99A	G283D	D264G	S19fs	1024	16	256

Genotypes and streptomycin resistances of reconstructed strains and the effects of *aadA* and *relA*, *spoT* inactivation on resistance. The symbol “*” represents a stop codon, while “fs” represents a frame shift mutation. MIC values presented are the median values of 2–5 Etests from biological replicates

Other types of mutations that were found in several of the sequenced clones were mutations in genes in the maltose operon (*malT* and *malF*) and genes encoding tRNAs. The *mal* mutations had no effect on resistance and are likely to be media adaptation mutations^31,32^. Similarly, mutations in genes involved in flagellar biosynthesis (*flgC*, *flgK*, *fliN*, and *fliJ*)^27^ were also seen in MH media adapted *Salmonella* strains, whereas the role of the tRNA mutations is unknown.

### The role of the *aadA* gene and the stringent response in resistance

Previous studies showed that activation of the cryptic aminoglycoside resistance gene *aadA* in *S*. *typhimurium* is ppGpp-dependent, and that upregulation of this gene results in increased resistance to streptomycin^25^. The *aadA* gene encodes an aminoglycoside adenylyl transferase which inactivates the drug by covalent addition of an adenylyl group. To examine the potential importance of *aadA* in the high-level streptomycin resistance observed in this study, we deleted the *aadA* gene in four of the clones isolated from the serial passage experiment (Table 1), as well as the reconstructed mutants (Table 2). This deletion resulted in a strong reduction of resistance for all mutants (Table 1), suggesting that the *aadA* gene has a central role in streptomycin resistance evolution at sub-MIC concentrations. Since the *aadA* gene is regulated by the stringent response, we also deleted the genes responsible for ppGpp synthesis, *relA* and *spoT*, and this also lowered the resistance levels of the majority of the strains (Tables 1 and 2). These findings demonstrate that the high-level streptomycin resistance of the strains require the ability to induce stringent response and presence of the AadA protein.

### Loss of *znuA* increase *aadA* transcript levels

**Fig. 3:**
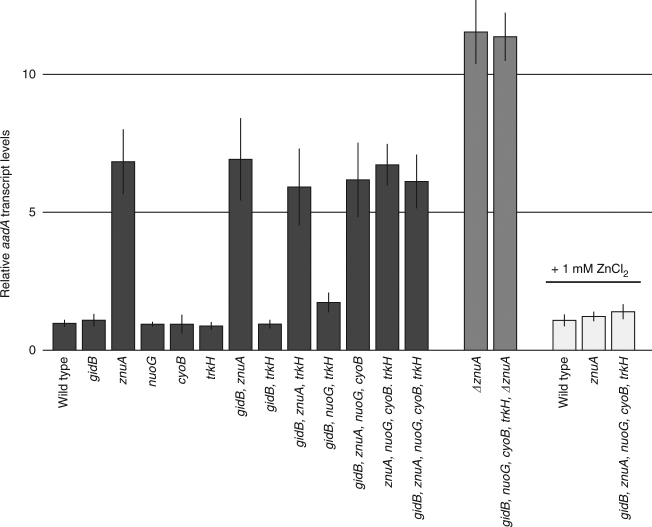
Relative *aadA* transcript levels. Transcript levels of the *aadA* gene were determined in reconstructed mutants (dark gray), *znuA* deletion mutants (gray), and reconstructed mutants grown in the presence of 1 mM ZnCl_2_ (white). Transcript levels were normalized to the housekeeping genes *cysG* and *hcaT* and all values are relative to transcript levels in the wild type. The error bars represent the standard deviation of two biological and three technical replicates each

Since high-level resistance requires the presence of the AadA protein (Tables 1 and 2), we determined if the increase in resistance in the reconstructed mutants was due to elevated *aadA* expression. We isolated total RNA at late exponential phase from all single mutants and two double, two triple, two quadruple, and the quintuple mutant and measured *aadA* transcript levels using qRT-PCR (Fig. 3). We observed a 7-fold increase in *aadA* expression in the *znuA* single mutant, while all other single mutants showed no significant increase. In accordance with this, all mutants with more than one mutation that also included *znuA* showed increased *aadA* expression levels to the same extent as the *znuA* single mutant. The two reconstructed clones containing multiple mutations without *znuA* (the double and triple mutants *gidB*, *trkH*, and *gidB*, *nuoG*, *trkH*) showed wild-type levels of *aadA* transcript. Thus, the *znuA* mutations (but not *gidB*, *trkH*, *cyoB*, or *nuoG* mutations) confer increased streptomycin resistance by causing *aadA* upregulation and inactivation of streptomycin via adenylylation. To test whether loss of ZnuA rather than the production of a frameshifted protein is causing the observed phenotype we constructed a deletion of the *znuA* reading frame in a wild-type background as well as in a quadruple mutant consisting of *gidB*, *nuoG*, *cyoB*, and *trkH* (Fig. 3). In both cases the deletion of *znuA* resulted in a 11-fold upregulation of *aadA* transcript levels, showing that loss of ZnuA is responsible for the observed phenotype. In addition, the upregulation of *aadA* due to loss of ZnuA was completely restored if the cells were grown in the presence of excess zinc, demonstrating that it is zinc starvation and not lack of the ZnuA protein per se that caused upregulation of AadA.

### *cyoB*, *nuoG*, and *trkH* mutations reduce streptomycin uptake

**Fig. 4:**
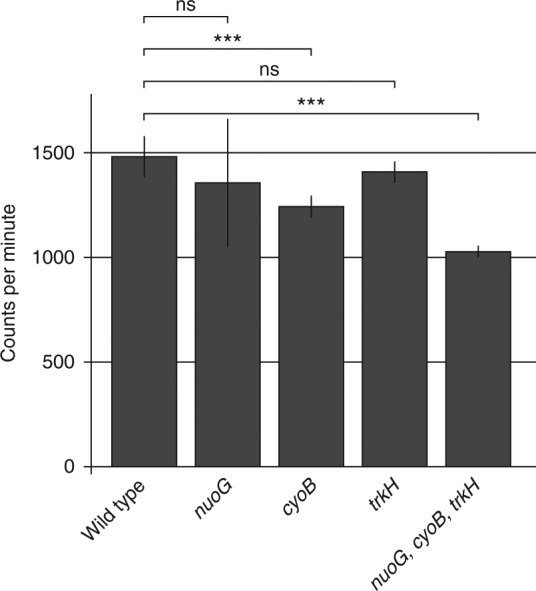
Intracellular uptake of H3-dihydrostreptomycin. Cells from late exponential phase were incubated 30 min in presence of 50 nCi ml^−1^ tritiated dihydrostreptomycin and washed to remove extracellular H3-dihydrostreptomycin. Counts per minute were determined for two biological and two technical replicates each. The error bars represent standard deviation. *t*-tests were performed to calculate the significance of the differences between mutants and wild type, where ns (not significant) indicates a *p-*value over 0.05 and three asterisks indicate high significance with *p-*values < 0.01

Since it is known that proton motive force is needed for streptomycin uptake^33–36^ and that the *cyoB*, *nuoG*, and *trkH* mutations are likely to impair electron transport^30,37^, we used radioactive streptomycin to measure if uptake was reduced in the mutants. Results show (Fig. 4) that one of the single mutants (*cyoB*) and the triple mutant (*trkH*, *nuoG*, *cyoB*) had a significant reduction in intracellular levels of tritiated dihydrostreptomycin, supporting the notion that these mutations confer increased resistance by impairing drug uptake. While the observed decrease in intracellular streptomycin is only moderate, streptomycin uptake and resistance level does not necessarily correlate in a linear and proportional way, and a small effect on uptake could conceivably have a large effect on resistance. Additionally, the reduction in streptomycin uptake is likely to be underestimated since the background signal in all strains might partly result from extracellularly bound streptomycin.

### Modeling the step-wise selection of five mutations

**Fig. 5:**
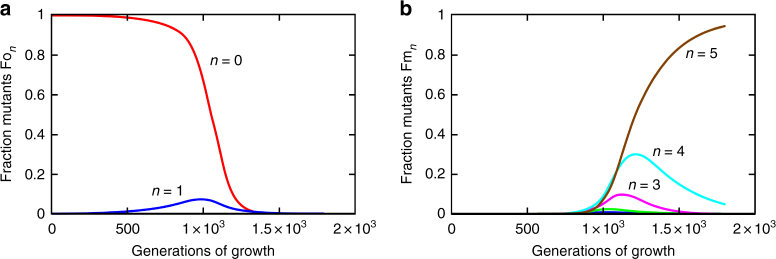
Population dynamics of different mutant types. Time development of the population fractions for *u* = 2 × 10^−6^ and *v*/*u* = 200. The fractions of the different variants over time in generations are shown on the *y*-axis, where Fo_*n*_ and Fm_*n*_ are the fractions of cells with *n* selected mutations for non-mutators and mutators, respectively. Non-mutators (**a**) contribute almost exclusively to *n* = 0 (red curve) and *n* = 1 (blue curve), while mutators (**b**) dominate totally in the fractions that carry three or more mutations; in the group with *n* = 2 (green curve), there is some mixing between mutators and non-mutators

To examine the dynamics of the step-wise enrichment of several mutations in one clone and determine under which conditions five step-wise mutations could accumulate in 900 generations under this weak selection, we modeled the selection process (for details, see Methods). In these calculations, we applied the experimentally determined population sizes and generations of growth used during serial passage. We also assumed that all selected mutations contribute independently to resistance/fitness and that each mutation has the same selection coefficient *s* = 0.006, such that five of them together give a 3% growth advantage in the presence of the antibiotic (during the selection the used concentration of streptomycin at ¼ MIC reduced the growth rate of the susceptible wild type by 3%). Furthermore, a mutator mutation is assumed to occur with the same rate, *u*, as each of the five selected resistance mutations (which are all loss-of-function mutations). In addition, it is assumed (as also observed experimentally) that the high-level-resistant mutants reached a frequency of 1% in the evolving population after 900 generations of growth. Results show that without mutators, 1% penetration after 900 generations can be achieved only with an unrealistically high mutation rate, *u* = 10^−3^, for each individual resistance mutation. In contrast, when mutators with mutation rate *v* are present, the mutant with five resistance mutations can reach 1% penetration in 900 generations within a realistic parameter range where mutation rate *u* varies ca. 10-fold between 5 × 10^−7^ and 4 × 10^−6^ and the ratio *v*/*u* also varies 10-fold between ca. 10^2^ and 10^3^ (Table 3, Fig. 5). Thus, even with a weak selection (*s* = 0.03) and only 900 generations of evolution, it is possible to step-wise accumulate five resistance mutations provided mutator clones appear early during serial passage.

**Table 3:** Penetration of five mutations Fo_5_ + Fm_5_ after 900 generations

*v*/*u*	90	125	203	285	400	460	1055
*u*							
5 × 10^−7^	5 × 10^−8^	2.5 × 10^−7^	2.8 × 10^−6^	1.5 × 10^−5^	8.3 × 10^−5^	1.7 × 10^−4^	**0.010**
1 × 10^−6^	3 × 10^−6^	1.6 × 10^−5^	1.8 × 10^−4^	9.4 × 10^−4^	5 × 10^−3^	**0.010**	0.27
2 × 10^−6^	1.9 × 10^−4^	9.5 × 10^−4^	**0.010**	0.048	0.17	0.24	0.65
3 × 10^−6^	0.002	**0.010**	0.083	0.24	0.43	0.5	0.76
4 × 10^−6^	**0.010**	0.045	0.23	0.42	0.57	0.62	0.81

Mutations in a non-mutator occur with the rate *u*, while the mutator cells have the mutation rate *v*. In this calculation, the mutation rate *u* has been varied between 5 × 10^−7^ and 4 × 10^−6^ and the ratio *v*/*u* between ca. 10^2^ and 10^3^. In these ranges, a 1% penetration of the mutant with five resistance mutations (indicated in bold) after 900 generations can be reached with a mutator rate *v* of ca. (3.5–5) × 10^−4^

## Discussion

In this study, we exposed susceptible wild-type bacteria to constant low levels of antibiotics (4-fold below the MIC, 1 mg L^−1^) for 900 generations, and found that high-level resistance (up to >1024 mg L^−1^) evolved in several independent lineages. By whole-genome sequencing and genetic reconstructions, we identified five causative resistance mutations of individual clones. The mutations had individually small effects, but when combined in a wild-type background they resulted in high-level resistance due to strong positive epistasis between certain mutations (Fig. 2, Supplementary Table 2). Since the evolved strains contained more mutations than the five that we studied in detail it is possible that other mutations also contributed to the resistance and fitness phenotypes.

An important question is how consecutive mutations in five different genes can accumulate over only 900 generations of selection with the weak selection used. Our modeling results show that with a 3% reduction in growth rate, a mutation rate for each consecutive mutation of around 3.5–5 × 10^−4^ per cell per generation is required for the step-wise accumulation of five consecutive mutations in 900 generations with the serial passage population sizes used and when the quintuple mutant constitutes 1% of the total population. All the resistance phenotypes are caused by loss-of-function mutations in the five different target genes and from previous work it is known that the average loss-of-function rate in a gene in a wild-type *E*. *coli*/*S*. *enterica* is approximately 10^−6^ per cell per generation^38,39^. In addition, all the examined clones are mutators, and the likely explanation for the mutator phenotype is that step-wise selection of several independent resistance mutations enriches for the mutators. It is well described that mutator alleles can rapidly become enriched in asexual populations during step-wise adaptation by second-order selection because they remain associated with the rare favorable resistance mutations they generate^14,40–43^. Importantly, the mutators (MutHLS mismatch repair system) found in this study exhibit an approximately 150-fold increase in mutation rate as compared to the wild type (Supplementary Table 3), which brings the expected loss-of-function for each individual gene to 10^−4^ per cell per generation, which is near the theoretically required mutation rate. We also attempted to experimentally determine which of the many potential routes to the high-level-resistant quintuple mutant (Fig. 1) occurred in the different lineages and how fitness changed in the evolving populations over time. However, this was not possible due to the fact that all of these strains are mutators and as a result the heterogeneity in each population is very high, making a time series analysis by deep sequencing of the evolving population difficult. In addition, the high heterogeneity in combination with the mutator phenotype also preclude us from making accurate measurements of small fitness differences (<1%) by competition since the competitor strains would rapidly acquire media adaptation mutations that alter their fitness.

**Fig. 6:**
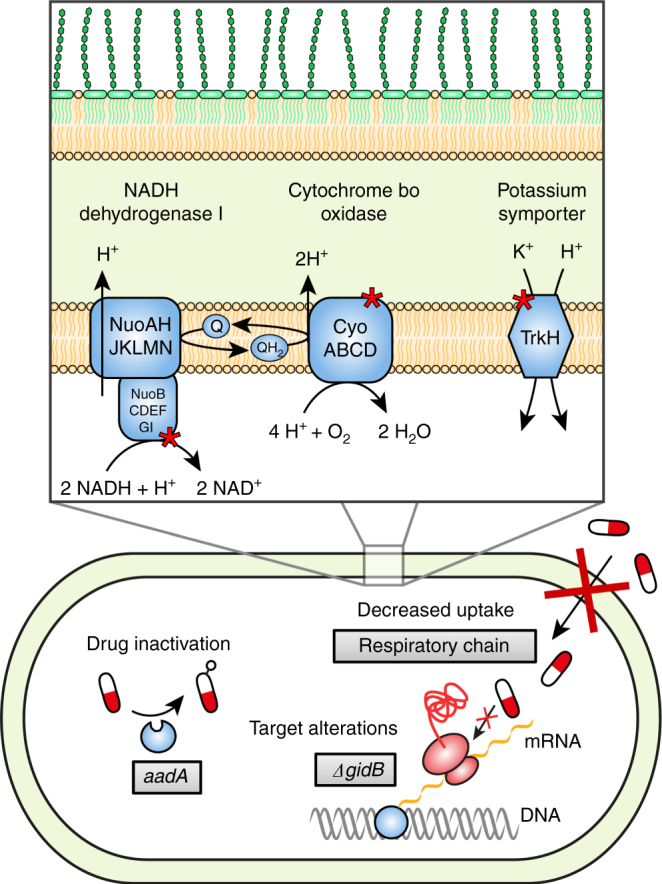
Three different resistance mechanisms that act in synergy. Increased expression of the aminoglycoside adenyl transferase gene *aadA* via inactivation of the gene *znuA* lowers the concentration of active drug through chemical modification, mutations in *gidB* indirectly modifies the drug target to decrease binding, and mutations in the respiratory chain lowers the membrane potential, causing a decrease in uptake of the drug

How is resistance conferred by the identified mutations? Based on the experiments presented here and previous studies, we propose that the high-level resistance is caused by the combined effect of three different mechanisms: (i) rRNA target alteration (due to *gidB* mutations), (ii) reduced aminoglycoside uptake (due to *cyoB*, *nuoG*, and *trkH* mutations), and (iii) induction of the aminoglycoside modifying enzyme AadA (due to *znuA* mutations) (Fig. 6). With regard to the first mechanism, all of the strains had loss-of-function mutations in the *gidB* gene, encoding a 16S rRNA methyltransferase. This enzyme methylates the 530 loop of 16S rRNA and if the modification is absent streptomycin binding to the ribosome is reduced^29^. Mutations in this gene have been reported to confer a moderate increase resistance to streptomycin in several species of bacteria, including *S*. *enterica*^25^ and *M*. *tuberculosis*^29^.

The second mechanism includes mutations that are located in genes encoding proteins involved in the electron transport chain, such as genes encoding subunits of NADH dehydrogenase (*nuoG*) and cytochrome o oxidase (*cyoB*). Aminoglycosides are cationic molecules, and they are believed to first penetrate the outer membrane through porins, and then cross the cytoplasmic membrane in a process that is dependent on electron transport through quinones and a sufficiently high membrane potential^35,36^. The mutations we observed in the respiratory chain (*cyoB* and *nuoG*) could potentially reduce electron transport and the membrane potential, and as result reduce streptomycin uptake. Similar mutations have also previously been found in streptomycin-resistant SCVs of *Salmonella*^24,25^, but while the SCVs have a very slow growing phenotype, the mutants selected in this study at sub-MIC levels of streptomycin have higher growth rates (Supplementary Table 3). Besides genes involved directly in the respiratory chain, the mutant with the highest level of streptomycin resistance also had a mutation in the potassium symporter TrkH. The gene encoding TrkH has previously been linked to streptomycin resistance, probably by affecting the membrane electrochemical potential^30,37^. Uptake assays with radiolabeled dihydrostreptomycin showed that, as expected, the *cyoB*, *nuoG*, and *trkH* mutants were impaired in drug accumulation, providing a conceivable explanation for how these mutations confer increased resistance (Fig. 4).

The third mechanism of resistance, enzymatic modification of streptomycin, is dependent on the expression of the cryptic aminoglycoside resistance gene *aadA*. Previous studies of *Salmonella* SCVs have shown that the *aadA* gene can be upregulated through the stringent response^25^, a stress response system that uses the global regulator ppGpp to control the distribution of resources between synthesis of ribosomes when nutrients are abundant, and the increased synthesis of metabolic enzymes during amino acid starvation^44^. Deleting the *aadA* gene in the mutants (both original and reconstructed) lowered resistance by 1.5-fold to 100-fold (Tables 1 and 2). However, after deletion of *relA* and *spoT*, which removes any ppGpp-dependent regulation^45^, strains carrying mutations in *gidB*, *cyoB*, *nuoG*, and/or *trkH* still show increased resistance to streptomycin compared to a *relA, spoT*-deficient wild-type strain indicating that their effect on resistance is not dependent on stringent response but on target modification via *gidB* and reduced uptake via *cyoB*, *nuoG*, and *trkH*. Only the effect of *znuA* mutations seemed to be abolished in a *relA*, *spoT*-deficient background (Table 2). In addition, we observed that the mutation in *znuA* alone was responsible for elevated *aadA* expression levels (Fig. 3). The periplasmic zinc binding protein ZnuA is part of the ZnuABC system. This system is of particular importance under zinc-limiting conditions, ensuring the high-affinity uptake of Zn^2+^^46^. Despite the fact that this system is repressed in the presence of adequate concentrations of zinc^47^ it is likely that basal levels are still expressed even during growth in rich medium like MH broth. Loss-of-function mutations in *znuA* are therefore likely to disrupt zinc homeostasis, an ion with a central role in essential cellular enzymes and processes^48^, and cause activation of cellular responses that subsequently could alter gene expression patterns. To determine if the observed upregulation of *aadA* in *znuA*-deficient mutants is mediated via increased ppGpp levels we measured the transcript levels of *iraP*, which is known to be positively regulated by elevated ppGpp levels^49^, in a subset of mutants. None of the tested strains showed an increase in *iraP* transcript levels indicating a ppGpp-independent mechanism even for *znuA* mutants. Furthermore, ppGpp levels were not consistently increased in the *znuA* mutants (Supplementary Fig. 1). The transcriptional changes caused by ppGpp are often augmented by the transcription factor DksA^50–52^. Since this protein contains a central Cys4 Zn-finger motif that is likely to be essential for correct folding and functioning^53,54^, we hypothesized that the observed effect of zinc depletion on *aadA* upregulation might be caused by the loss of functional DksA. However, deletion of *dksA* in a wild-type background did not cause upregulation of *aadA*. Additionally, the introduction of a zinc-independent *dksA* paralog from *Pseudomonas aeruginosa*^55^ in a *znuA* mutant did not restore wild-type levels (Supplementary Fig. 2), showing that misfolding of DksA under zinc starvation is not responsible for the observed phenotypes. While the exact mechanism of *aadA* upregulation via *znuA*-mediated zinc-depletion remains unclear, our results suggest that it is not mediated via ppGpp or DksA, but requires functional RelA and SpoT, possibly due to a dominant regulatory effect exerted by these proteins.

What are the general implications of this work? First, and most importantly, our results demonstrate that very weak and constant selective pressures of antibiotics can result in the evolution of high-level resistance. Thus, an implication from these results is that there is no necessary correlation between strength of selection and level of resistance evolved, and as a consequence it is conceivable that the low antibiotic levels present in many environments could drive the evolution of clinically relevant high-level resistance. Second, our data show that mutational spectra can differ strongly between different selective strengths. Thus, at lethal selections only *rpsL* mutations are found, whereas at sub-MIC selections a different set of mutations are selected. A likely explanation for this difference is that at >MIC levels of drug only *rpsL* mutations provide a sufficiently high level of resistance to be enriched, whereas at low concentrations the more common mutations in the genes identified here are preferentially observed (even though *rpsL* mutations occur but at a much lower rate). Third, these results show that the high-level resistance can result from many different mutations of small individual effect that show positive epistasis. Thus, among the five genes examined only mutations in the *gidB* gene conferred measurable resistance by itself, whereas when the five mutant genes were combined resistance reached very high levels. Furthermore, a majority of these mutations were in genes that are not typical resistance genes (i.e., *cyoB*, *nuoG*, *trkH*, and *znuA*). This finding raises the question if a substantial “dark matter” of unknown resistance mutations for other classes of antibiotics exists in clinical isolates that apart from the generally easily identified bona fide resistance genes/mutations may be involved in resistance evolution and make a significant contribution to the clinical resistance phenotype^14,18,19,56^. Thus, it is conceivable that the initial selection for common mutations of small effect could act as a stepping stone for the subsequent occurrence of a rarer large effect genetic event (e.g., horizontal gene transfer of a bona fide resistance gene) and thereby act as a driver of resistance evolution. Also, considering that individually the mutations in *cyoB*, *nuoG*, *trkH*, and *znuA* conferred no/minor increases in resistance, these types of atypical resistance mutations would be difficult to identify in clinical isolates using standard genetic procedures and reconstruction. That is, transfer of DNA from a resistant strain by transformation/transduction to a susceptible strain to identify potential resistance mutations is unlikely to work since the individual mutations except for *gidB* had minor effects on resistance. Instead, the clinical identification of these types of mutations would first require laboratory selection and reconstitution experiments (as done here) for identification, and then a subsequent comparative analysis of susceptible and resistant clinical isolates to search for their potential presence in resistant isolates. Finally, it is worthwhile noting that the potential occurrence of atypical resistance mutations in clinical isolates would make sequence-based in silico predictions of antibiotic resistance more challenging.

## Methods

### Strains and growth conditions

All strains used in this study were derived from *Salmonella enterica* serovar Typhimurium LT2 (designated *S*. *typhimurium* in the text) and are listed in Tables 1 and 2, and Supplementary Tables 4 and 5. The liquid and solid media used for bacterial growth were MH broth (Becton Dickinson, MD, USA), MH agar (MH broth supplemented with 1.5% agar), and Luria–Bertani (LB) agar (Sigma-Aldrich, MO, USA). Strains were grown at 37 °C, and liquid cultures were aerated by shaking.

### Selection for resistance above MIC

Selection of *S*. *typhimurium* mutants with decreased susceptibility to streptomycin was performed by plating 10 independent cultures (each inoculated with 10^3^ cfu) of the wild-type strain DA6192 on MH agar containing 200 mg L^−1^ streptomycin (Sigma-Aldrich). Approximately 10^10^ cfu from each culture were plated. The mutants (one per independent culture) were randomly picked after 24 h of incubation at 37 °C and re-isolated on plates with the same concentration of streptomycin. Cultures of isolated clones were grown over night at 37 °C in MH broth without selection and frozen in −80 °C with 10% sterile DMSO. The *rpsL* gene in the mutants was PCR amplified with primers binding upstream and downstream of the coding region using DreamTaq DNA polymerase (Thermo Scientific). Populations of *S*. *typhimurium* were also evolved in presence of high levels of streptomycin in liquid medium by passaging 10 lineages of the wild-type strain DA6192 in MH medium with 200 mg L^−1^ streptomycin. The lineages were started from overnight cultures of independent colonies grown in MH without selection. The evolution experiment was initiated with an inoculum size of approximately 10^10^ cells. The cultures were serially passaged by 1000-fold dilutions in 1 ml batch cultures every 24 h, and after 100 generations of cycling the cultures were frozen in −80 °C with 10% sterile DMSO. Six mutants selected on solid media and five populations evolved in liquid media were whole-genome sequenced to identify possible streptomycin resistance mutations outside of *rpsL* (Supplementary Table 4).

### Laboratory evolution of resistance at sub-MIC

The evolution of de novo resistance has been described previously^8^. Briefly, bacteria were grown in MH medium with 1 mg L^−1^ of streptomycin for 24 h at 37 °C with shaking and then 1 µl culture was transferred generating a maximum and minimum population of 5 × 10^9^ ml^−1^ and 5 × 10^6^ ml^−1^, respectively, during each step of the serial passage. After 900 generations (approximately 90 serial passages) of cycling, 10^5^ cells were plated onto LB agar containing different concentrations of streptomycin (8, 16, 32, 64, 96, 128, 192, and 256 mg L^−1^), and 2–3 resistant clones were isolated from each lineage where they represented a fraction of the population of approximately 1% (Supplementary Table 5). The isolated clones were frozen in −80 °C in 10% DMSO, and the evolving lineages were frozen every 100 generations.

### Whole-genome sequencing

Genomic DNA (gDNA) was extracted from 4 ml of overnight cultures of the mutants isolated after 900 generations of growth in 1 mg L^−1^ of streptomycin using the Genomic-tip 100/G columns and the Genomic DNA Buffer Set (Qiagen, The Netherlands), according to the manufacturer’s instructions. Quality of gDNA were examined using NanoDrop spectrophotometer (Thermo Scientific, Waltham, MA, USA) and separated by electrophoresis on 0.8% (w/v) agarose gels for visualization. The gDNA was sent to the Beijing Genomic Institute sequencing facility (Hong Kong, China) for whole-genome resequencing using the Illumina HiSeq 2000 sequencing system (Illumina Inc., San Diego, CA, USA) with 50× coverage. Briefly, sequencing libraries per gDNA were prepared according to the Solexa sequencing protocols (Illumina Inc., San Diego, CA, USA). Illumina analysis pipeline was used for image analysis, base calling, and quality score calibration. Subsequently, raw sequence reads were quality filtered and exported as FASTQ files resulting in 12,000,000 high-quality sequence reads per gDNA isolated. For the clones and populations selected with 200 mg L^−1^ of streptomycin, DNA extraction was done from 1 ml of overnight cultures using Epicentre MasterPure Complete DNA and RNA Purification Kit (Illumina Inc., San Diego, CA, USA) following the manufacturer’s instructions. Libraries to be sequenced with Miseq (2 × 300) were made using the Illumina’s Nextera XT kit, and samples were dual-indexed and pooled. The paired-end sequence reads have been deposited to the NCBI Sequence Read Archive (SRA) with accession no. SRP133288.

The reads were mapped to the previously sequenced *S*. *typhimurium* DA6192 in house reference genome^27^ using CLC Genomics Workbench version 11 (Qiagen Bioinformatics, Aarhus, Denmark) with default parameters (masking mode = no masking; match score = 1; mismatch cost = 2; cost of insertions and deletions = linear gap cost; insertion cost = 3; deletion cost = 3; length fraction = 0.5; similarity fraction = 0.8; global alignment = no; auto-detect paired distances = yes; non-specific match handling = map randomly). Subsequently, InDels and structural variants were determined following default parameters (*p*-value threshold = 0.0001; maximum number of mismatches = 3; create breakpoints = no; ignore broken pairs = yes; minimum relative consensus coverage = 0.0; minimum quality score = 0; filter variants = no; minimum number of reads = 2; restrict calling to target regions = not set), and single-nucleotide polymorphisms were determined following default parameters (ploidy = 2; ignore positions with coverage above = 100,000; restrict calling to target regions = not set; ignore broken pairs = yes; ignore non-specific matches = reads; minimum coverage = 2; minimum count = 2; minimum frequency (%) = 75.0; base quality filter = yes; neighborhood radius = 5; minimum central quality = 20; minimum neighborhood quality = 15; read direction filter = yes; direction frequency (%) = 5.0; relative read direction filter = yes; significance (%) = 1.0; read position filter = no; remove pyro-error variants = no).

### Sanger sequencing

Mutated regions were PCR amplified with primers binding upstream and downstream of the mutation using DreamTaq DNA polymerase (Thermo Scientific). PCR products were purified and sent to Eurofins MWG Operon (Ebersberg, Germany) for sequencing.

### Strain reconstruction

The reconstructed strains were constructed by using Duplication-Insertion Recombineering^57^ and P22 transduction. A *cat-sacB* marker (GenBank accession number KM018298) was inserted near each mutation using Lambda-Red recombineering^58^, generating a marker-held tandem duplication. The marker and the linked mutation were then moved using P22 transduction into the recipient strains, and the transductants were re-streaked on salt-free LB agar containing 5% sucrose. Only bacteria that lose the *cat-sacB* marker through recombination between the two copies of the duplicated sequence can grow since expression of *sacB* is lethal in the presence of sucrose^59^. The transduced mutations were then confirmed by PCR and sequencing. Gene deletions were constructed by replacing the gene with a *cat-sacB* cassette, and then removing the cassette with a single-stranded DNA oligo in a second round of recombineering. Primer sequences are listed in Supplementary Table 6. Whole-genome sequencing showed that some of the reconstructed strains carried additional, unintended mutations (Supplementary Table 7). Two of them, *yebB* (S54G) and *lon* (P12syn), were co-transduced from the isolated mutants, while the rest appeared during the reconstruction process.

### Growth rate measurements

Growth rates were measured at 37 °C in MH broth using a Bioscreen C Analyzer (Oy Growth Curves Ab Ltd., Helsinki, Finland). Each well was inoculated with a 1000-fold dilution of an overnight culture and measurements were made in quadruplicates. The cultures were grown for 24 h with continuous shaking, and OD_600_ measurements were taken every 4 min. The calculations were based on OD_600_ values between 0.02 and 0.1, where growth was observed to be exponential.

### MIC measurements

MIC values were determined by Etest according to the instructions of the manufacturer (bioMérieux, Marcy l'Étoile, France). Etests were performed on MH agar plates incubated for 16–18 h at 37 °C. All MIC values presented are the median values of 2–5 Etests from biological replicates.

### Spontaneous mutation rates

The mutation rates of the different strains were estimated with fluctuation assays^60^. From frozen stock, the strains were streaked on MH agar plates and single colonies were used to start overnight cultures in 1 ml MH medium. These cultures were diluted 10^3^ in sterile filtered phosphate-buffered saline and 24 independent cultures of each strain were started with an initial inoculum of approximately 1000 cfu in 1 ml MH medium. The cultures were incubated in 37 °C for 20 h to stationary phase, and rifampin-resistant mutants were detected by plating a fraction of each culture on MH agar medium supplemented with 100 mg L^−1^ rifampicin. The volumes plated was 50 µL for non-mutator strains and 5 µL for mutator strains. The population sizes of the cultures were estimated by plating on non-selective MH agar plates. Plates were incubated for 24 h in 37 °C before counting the number of colonies per plate. The estimated mutation rates were calculated with the empirical probability generating function using the web tool bz-rates^61^, with correction for the plating efficiency.

### Isolation of total RNA and qRT-PCR

Overnight cultures were diluted 1:100 in 10 ml MH broth and incubated at 37 °C to OD_600_ = 0.4. To test the effect of excess zinc on *aadA* expression some cultures were supplemented with 1 mM ZnCl_2_. 400 µl of bacterial culture were mixed with 800 µl RNAprotect Bacteria Reagent (Qiagen) and total RNA was extracted using the RNeasy Mini Kit (Qiagen), according to manufacturer’s recommendation. Subsequently, the extracted RNA was treated with DNase Turbo DNA-free kit (Amb ion) according to manufacturer’s recommendation and subjected to gel electrophoresis to confirm the integrity of the samples. 500 ng of DNase-treated RNA were reverse transcribed using the High Capacity Reverse Transcription kit (Applied Biosystems), according to the manufacturer’s recommendation. Relative *aadA* and *iraP* transcript levels were determined using PerfeCTa SYBR Green SuperMix (Quanta Biosciences) with the Eco Real-Time PCR System (Illumina). The efficiency of each primer pair was determined in a set of six 10-fold dilutions. Expression levels of *aadA* and *iraP* were normalized to the reference genes *hcaT* and *cysG*. Primer sequences are listed in Supplementary Table 8. Measurements were repeated for at least two biological and three technical replicates each.

### Streptomycin uptake assay

To determine the concentration of intracellular dihydrostreptomycin an overnight culture of the strain of interest was diluted 1:200 in MH broth and grown to OD_600_ = 0.5. The cultures were supplemented with tritiated dihydrostreptomycin (Larodan, Sweden) to a final activity of 50 nCi ml^−1^. After 30 min, 2 ml were transferred to an Eppendorf tube, washed three times with PBS and finally transferred to 2 ml Optiphase HiSafe 3 (PerkinElmer) scintillation cocktail. Counts per minute were determined using a Tri-Carb 2810 TR liquid scintillation analyzer (PerkinElmer) over a duration of 1 min per sample.

### Model for mutation accumulation and takeover

We consider a deterministic growth model where back mutations and stochastic effects are neglected. Let *N*_*n*_(*t*) be the number of cells with *n* selected (by *s* each) mutations at time *t*, and *M*_*n*_(*t*) be the corresponding number of mutator cells. They grow with rate (1 + *ns*)*k*. Up to five selected mutations can occur in random order each with the basic rate *u*. Mutators are also assumed to occur with rate *u*. When *n* selected mutations are present, the next one can occur in 5-*n* ways; hence with total rate *u*(5 − *n*)(1 + *ns*)*k*. The mutator cells have the basic mutation rate *v*.

1 $$\frac{{{\mathrm{d}}N_n}}{{{\mathrm{d}}t}} = \left( {1 + ns} \right)kN_n + u\left( {6 - n} \right)\left[ {1 + \left( {n - 1} \right)s} \right]kN_{n - 1} \hfill\\ \frac{{{\mathrm{d}}M_n}}{{{\mathrm{d}}t}} = \left( {1 + ns} \right)kM_n + v\left( {6 - n} \right)\left[ {1 + \left( {n - 1} \right)s} \right]kM_{n - 1} + u\left( {1 + ns} \right)kN_n.$$

With *N*_-1_ and *M*_-1_ = 0 at all times, these equations are valid for $0 \le n \le 5$. The initial conditions are:

2 $$\begin{array}{*{20}{l}}N_0\left( 0 \right) & = N_{\rm e} \hfill\\ N_1\left( 0 \right) & = 0\ {\mathrm{or}}\,N_1\left( 0 \right) = N_{\rm e} 5u/s\\ N_n\left( 0 \right) & = 0;\,2 \le n \le 5 \\ \hskip -2.6pc M_n\left( 0 \right) & = 0;\,0 \le n \le 5\end{array}$$

Here, *N*_*e*_ is an effective population size. A non-zero initial value for *N*_1_(0) is based on the equilibrium value in a population without antibiotics where a mutation will be counter-selected by –*s*. The solution for the non-mutator part of the population is

$$N_0\left( t \right) = N_{\rm e} {\rm e}^{kt}$$

$$N_n\left( t \right) = N_{\rm e}{\rm e}^{\left( {1 + ns} \right)kt}\left( {\begin{array}{*{20}{c}} 5 \\ n \end{array}} \right)\left( {\frac{u}{s}} \right)^n\mathop {\sum }\limits_{j = 0}^{n - 1} \left( {1 + js} \right)\left[ {\left( {1 - {\rm e}^{ - skt}} \right)^n + An\left( {1 - {\rm e}^{ - skt}} \right)^{n - 1}} \right];$$

3 $$1 \le n \le 5$$

The parameter *A* = 0 is for the case with *N*_1_(0) = 0 and *A* = 1 corresponds to the case where *N*_1_(0) = *N*_*e*_5*u*/*s*. The mutators are most easily handled by numerical integration. Let

$$M_n\left( t \right) = {\rm e}^{\left( {1 + ns} \right)kt}g_n\left( t \right)$$

Then

$$g_0\left( t \right) = N_{\rm e}ut$$

$$\frac{{{\mathrm{d}}g_n}}{{{\mathrm{d}}t}} = v\left( {6 - n} \right)\left[ {1 + \left( {n - 1} \right)s} \right]k{\rm e}^{ - st}g_{n - 1}\left( t \right) + u\left( {1 + ns} \right)k{\rm e}^{ - \left( {1 + ns} \right)kt}N_n\left( t \right){\mathrm{;}}$$

4 $$1 \le n \le 5$$

The fractions of the different variants at any time can be calculated from

5 $${\rm Fo}_n = N_n{\mathrm{/}}\mathop {\sum }\limits_n \left( {N_n + M_n} \right)\;{\mathrm{and}}\;{\rm Fm}_n = M_n{\mathrm{/}}\mathop {\sum }\limits_n \left( {N_n + M_n} \right).$$

In the calculations, it has been assumed that all selected mutations contribute independently and that each has the same selection coefficient *s* = 0.006, such that five of them give a 3% growth advantage in the presence of the antibiotic. Furthermore, a mutator mutation is assumed to occur with the same rate, *u*, as each of the selected ones. It is straightforward to expand the calculations to include a non-zero initial presence of mutators. However, with an expected fraction on the order of 10^−6^, this is found to have a very small effect on the results and is not considered further. The expected initial fraction of single mutants, 5 *u*/*s*, may be on the order of 10^−3^, which can contribute significantly. Variants with two or more mutations are expected to be present in insignificant fractions initially. Mutators are expected to accumulate deleterious mutations over time contributing to some growth disadvantage for them. Here it is assumed that this effect is small on the time scale considered. Finally, the growth rate *k* = ln(2) if time, *t*, is considered in number of generations (doublings). Figure 5 summarizes the time development of the different mutant types.

**Fig. 7:**
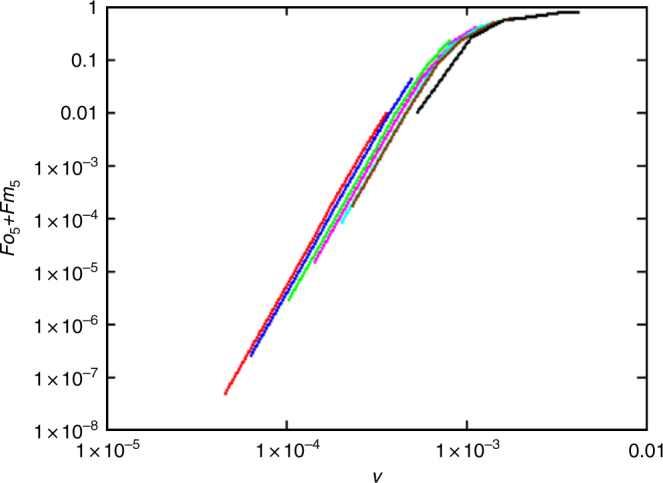
Penetration of mutants with five selected mutations. Penetration after 900 generations as a function of mutator mutation rate *v* for values of *u* between 5 × 10^−7^ (black curve on the right) and 4 × 10^−6^ (red curve on the left)

Without mutators, 1% penetration after 900 generations can be achieved only with a very high mutation rate, *u* = 9.5 × 10^−4^, or slightly lower, *u* = 7.4 × 10^−4^, if there is a fraction 5 *u*/*s* of single mutants present initially. When mutators contribute, this initial condition has little effect and has not been included in the calculations of the results shown in Fig. 7 and Table 3. We have varied the mutation rate *u* ca. 10-fold between 5 × 10^−7^ and 4 × 10^−6^ and the ratio *v*/*u* also 10-fold between ca. 10^2^ and 10^3^. It is noteworthy that throughout these ranges, 1% penetration is reached with a mutator rate *v* of ca. (3.5–5) × 10^−4^.

### Calculating epistasis using the weighted Walsh–Hadamard transform

We studied five different genetic loci with either the wild-type or a mutant sequence, making a total of 2^5^ = 32 genotypes, so the collection of all 32 reconstructed mutants with resistance phenotypes (Fig. 1 and Table 2) represents a combinatorially complete dataset for the five mutations. The fitness landscape can be seen as a high-dimensional space where different orders of epistatic (non-additive) interactions can be calculated through spectral analysis using transform theory^62,63^, similarly to how a Fourier transform can be used to decompose a signal in the time domain into different components in the frequency domain. The higher-order epistatic effects of the different resistance mutations were calculated through Walsh–Hadamard transform decomposition^64^ of the fitness landscape of streptomycin resistance. Since this is a linear epistasis model while antibiotic resistance increases in a multiplicative way, the phenotypic resistance values were linearized as log_2_(MIC).

The epistatic effects were calculated in two different ways; as the global epistatic effect of each mutation (background-averaged epistasis, corresponding to a Fourier expansion), and as the epistatic effect of each mutation with the wild-type sequence as reference (corresponding to a Taylor expansion)^64^. Interactions between the different mutations that are purely additive will have epistatic coefficients equal to zero, while epistatic interactions will be non-zero. The epistasis coefficients of each order are calculated so that higher-order epistasis coefficients only represent the variation that cannot be described as the sum of the lower-order effects. The mathematical theory behind the model has been thoroughly discussed by Poelwijk et al.^64^, so we will only give a summary of the model here.

If we define ${\bar{\bf{\omega }}}$ as the vector of 2^*n*^ epistasis coefficients of all orders, ${\bar{\boldsymbol y}}$ as the vector corresponding to phenotypes *y* (log_2_(MIC)) of all the individual variants listed in binary order (Supplementary Table 2), and the 2^*n*^ × 2^*n*^ matrix ${\mathbf{\Omega }}_{{\mathrm{epi}}}$ as the epistasis transform, we can write

6 $${\bar{\bf{\omega }}} = \Omega _{{\mathrm{epi}}}{\bar{\boldsymbol y}}.$$

We first calculated the global, background-averaged epistasis *ε*; the epistatic effects of each mutation as an average over all possible sequence variants. In this case, we can define

7 $${\bf{\Omega }}_{{\mathrm{epi}}\_{\mathrm{global}}} = {\boldsymbol{VH}},$$

where ***V*** is a weighting matrix to normalize for the different numbers of terms for epistasis of different orders, recursively defined as

8 $${\boldsymbol{V}}_{n + 1} = \left( {\begin{array}{*{20}{c}}{\frac{1}{2}{\boldsymbol{V}}_n} & 0 \\ 0 & {{\boldsymbol{V}}_n} \end{array}} \right){\mathrm{with}}\;{\boldsymbol{V}}_0 = 1$$

and the matrix ***H*** corresponds to the Walsh–Hadamard transform^63,64^ generated by the recursive definition

9 $${\boldsymbol{H}}_{n + 1} = \left( {\begin{array}{*{20}{c}} {{\boldsymbol{H}}_n} & {{\boldsymbol{H}}_n} \\ {{\boldsymbol{H}}_n} & { - {\boldsymbol{H}}_n} \end{array}} \right)\,{\mathrm{with}}\,{\boldsymbol{H}}_0 = 1.$$

The vector of background-averaged epistatic terms ${\bar{\bf{\varepsilon }}}$ can then be calculated by

10 $${\bar{\bf{\varepsilon }}}{\mathrm{ = }}{\boldsymbol{VH\bar y}}.$$

To calculate the vector of epistatic terms ${\bar{\boldsymbol \lambda }}$ relative to the wild-type sequence, we instead use

11 $${\bf{\Omega }}_{{\mathrm{epi}}\_{\mathrm{wt}}} = {\boldsymbol{VX}}^{\boldsymbol{T}}{\boldsymbol{H}},$$

where ***X***^***T***^ only includes the terms associated with the wild type, and the matrix ***X*** can be defined as

12 $${\boldsymbol{X}}_{n + 1} = \left( {\begin{array}{*{20}{c}} {{\boldsymbol{X}}_n} & 0 \\ {{\boldsymbol{X}}_n} & {{\boldsymbol{X}}_n} \end{array}} \right)\,{\mathrm{with}}\,{\boldsymbol{X}}_0 = 1,$$

which gives us

13 $${\bar{\boldsymbol \lambda }} = {\boldsymbol{VX}}^{\boldsymbol{T}}{\boldsymbol{H\bar y}}.$$

The calculations were performed using Python 3.6 with the NumPy library.

### Code availability

Python scripts are available from the corresponding author upon request.

### Data availability

All WGS data that support the findings of this study have been deposited in the NCBI SRA and are accessible through the accession no. SRP133288. All other relevant data are available from the corresponding author upon request.

## Electronic supplementary material

Supplementary Information

Peer Review File
