# Supplementary material for: Evolution of high-level resistance during low-level antibiotic exposure
Source: Nat Commun. 2018 Apr 23;9:1599. doi: 10.1038/s41467-018-04059-1 (PMC5913237; doi:10.1038/s41467-018-04059-1)
Supplement: Supplementary file 1 — Supplementary Information [file 41467_2018_4059_MOESM1_ESM.pdf]

Reviewers' comments:

Reviewer #1 (Remarks to the Author):

The manuscript submitted to Nature Communications by Wistrand-Yuen et al. reports how high-level streptomycin resistance emerges in response to low doses of streptomycin in salmonella enterica. In the abstract and in the introduction, authors report their main finding as "These results demonstrate how the strength of the selective pressure influences evolutionary trajectories and that even very weak selective pressures can cause evolution of high-level resistance." Although this is an important argument, the data provided by the authors do not provide much evidence to support that. Besides, this finding is not novel and has been previously reported by others. I have several comments about the manuscript and unfortunately I do not think that the manuscript is suitable for publication at this stage.

1. The introduction and the abstract emphasize the importance of selection in low doses of antibiotics. I agree with the authors about the importance of this issue. Indeed, regardless of how antibiotics are utilized, there will always be residual (intact or degraded) antibiotic molecules in the environment and this may select for resistance. However, the choice of antibiotic dosage for the laboratory evolution experiments for this is study is not justified. 25% of the MIC was used for the selection and authors report that this causes only ~3% growth rate reduction for the wild type ancestor strain. I do not understand how such a small change can be responsible for evolution of high levels of resistance and emergence of mutator strains (4 out of 10). Growth rate data for salmonella in different streptomycin concentrations are not provided therefore it is difficult for me to understand (predict) other changes caused by 1mg/l streptomycin. Growth rate is not the only phenotype that should be monitored for fitness. The lag time or the yield can change as well. For example, mutations that reduce the lag time in low dose antibiotics will certainly be selected. Besides, since fitness measurements were based on OD600 reads, corresponding cfu/ml counts could be useful to understand the level of selection by the drug dose used.

2. One of the major claims of this work is the existence of rpsL mutations in only strong selection. I have read the relevant parts of the paper several times to clarify and I might still be missing something. I apologize in advance if this is due to my lack of understanding. According to the materials and methods section, the assay for strong selection was done using 24 hours of growth in 200mg/l streptomycin on solid agar, whereas the weak selection was done in small volumes of well shaken liquid media for 900 generations. If this is correct, this is not a fair comparison to begin with. It is known that resistant colonies selected on solid agar will not have multiple mutations. Typically, one mutation will be enough to survive. However, in liquid media, several sub-populations will compete and multiple mutations will emerge. This comparison is not fair since the drug concentration is not the only variable.

3. My understanding is that only 4 (highly resistant) clones from weakly selected populations were sequenced by whole genome sequencing. I honestly could not understand whether clones selected in high doses were whole genome sequenced. If I am not wrong, only the rpsL gene was sequenced by Sanger sequencing. Therefore, it is not possible to rule out the existence of other mutations in strongly selected clones.

4. Authors chose 4 most resistant clones and whole genome sequenced them. I certainly understand that there might be financial constraints limiting the number of sequenced clones. However, I am a bit concerned that all of the sequenced strains turned out to be mutators. The remaining resistant clones may or may not be mutators and their genotypes are not known if I am not missing something. It was

not possible to understand this from table S1 and the text. (i) they might have mutations in rpsL; (ii) they might not be mutators; (iii) they might have only single mutations. Therefore, the conclusions about the genetic pathways and mechanisms are not well supported by the sequencing data.

5. Examining the effects of five mutations in all possible combinations and examining the effect of aadA regulation is certainly very useful for everyone in the field. However, it does not add much to the main claims of the paper.

6. The epistasis calculations can be done more systematically. The combined epistasis term is shown in figure 2. It clearly suggests that there is epistasis but it can be more informative if epistatic terms were calculated depending on their orders (1st order, 2nd order, 3rd order, 4th order). One can use the formalism described by Poelwijk et al. (Poelwijk FJ, PLoS Comput Biol 12(6): e1004771.). https://doi.org/10.1371/journal.pcbi.1004771. Also, using a log scale on the y axis of fig 2 might be helpful.

7. Fig.4. Reduction in the intracellular uptake of streptomycin does not seem to be large enough to explain elevated streptomycin resistance. Since all of these strains were already phenotyped for resistance, it should be straightforward to calculate the correlation.

Reviewer #2 (Remarks to the Author):

Review Wistrand-Yuen et al- Nature Communications 2017

This is a very interesting and well written manuscript demonstrating how selection strength influence the evolutionary trajectories of antibiotic resistance. Whereas this has been indicated in previous work, this paper the first to address this mechanistically through elegant reconstructions of combinations of all mutations predicted to play a role in antibiotic (streptomycin) resistance.

The authors present a "fitness landscape" of all possible combinations of five mutations predicted to play a role in evolution of streptomycin resistance. Interestingly, whereas only one of these mutations significantly elevated resistance alone, the combination of all five mutations resulted in high level streptomycin resistance through the combined effects of three different mechanisms (target modification, reduced uptake and induction of a chromosomal aminoglycoside modifying enzyme). The authors present compelling evidence of strong positive epistasis between individual resistance mutations of low effect. The emergence of several mutations with reduced fitness (without immediate benefit) during 900 generations of experimental evolution is rationalized by a mathematical model.

This is solid work with properly controlled experiments and it is of great interest to a broad scientific audience. The work increases our understanding of how low concentrations of antibiotics may select for high level resistance (i.e. clinically relevant) through a remarkable genetic repertoire of atypical resistance mutations. This paper will, if my points below are addressed adequately, significantly influence thinking in the field.

Critical points:

1. Whereas I do not doubt that resistance increases stepwise with combinations of several of these

mutations Table 2 displays several intermediate MIC values Please include a description of how the MICs were determined with respect to a) number of experiments and b) if MICs were modes or averages of several repeated experiments. Several intermediate MICs are reported as values below 2-fold changes. This is arguably problematic since clinical laboratories often require 4- fold changes in both directions in order to report significant changes in susceptibilities. However, if the values are based on multiple biological replicates this point can be ignored.

Please go over Table 2 and correct txt, or table- several inconsistencies:

2. P 7, line 3: gidB mutation MIC = 48 mg/L – Table 2 reports 32 mg/L.
3. P 7, line 4: znuA is reported to result in 2- fold change- listed in Table 2 as 1,5 fold change- significant?
4. P7, line 11: gidB + both respiratory chain mutations is listed as MIC of 256 mg/L, and not 192 in Table 2.
5. P7, line 13: quadruple mutant MIC og 192- right?
6. P8, line 16: Table 2 lists 7 cases, and Table 1 a single case, where no reductions in MIC were reported following deletion of relA and spoT
7. P13, line 24: 50-100 fold decrease: this does not reflect the numbers reported in Tables 1 and 2- please correct.
8. P15 and 16 lines 24 and 1, respectively. Technically this may be true- I believe the authors mean downregulation of aadA- right?
9. The astute reader will observe that the combination of gidB and cyoB alone (Strain DA23872) gave a MIC of 512 mg/L. In table 1, the reconstruction show that the presence of these 2 mutations gave an MIC of 96 mg/L. The authors might consider a short elaboration of the possibility that many more mutations of small effects may contribute to high level resistance.

Other points:

10. Lack of proper fitness estimates is well explained in the discussion. However, the Lenski lab (and others) have reported in a series of papers competitions with mutator E. coli evolved strains and populations. I assume that the problem here is the small-fitness effects of individual mutations and that longer time series needed to accurately measure these are hampered by deviations from linearity in the competition experiments and that shorter competition experiments did not result in sufficient resolution- could this be worth explicitly stating?
11. Model: I find it slightly under explained- whereas it is mentioned in the discussion that the rather narrow bottlenecks are included (Ne component) I believe it would benefit readers that hums rather than solves equations to explicitly explain this.

- The manuscript would also benefit from a parameter-table.
- Table 3 needs to be better explained- it is difficult to understand (at least the version I got for review).
- Figure 5 would also benefit from a revision where Y- axes are clearly explained and the Fig txt should be fully explanatory

12. Concentration of sucrose used is missing in material and methods
13. Concentrations of streptomycin on plating assays
14. Supp Table 2: Explain DIP (deletion insertion polymorphisms?)
15. * on Q169 mutation (Table 2) (stop codon?)

Reviewer #3 (Remarks to the Author):

The authors present a comprehensive analysis of the evolutionary landscape of Salmonella Typhimurium when exposed to sub-MIC levels of streptomycin. The level of molecular biology and mechanistic research undertaken is somewhat of a tour-de-force, and the findings represent novel and important conclusions for all working on AMR.

However I have a few points I would like to raise with the authors in the interest of possibly strengthening the presented manuscript.

Major issues:
An obvious question that immediately came to my mind is why use Salmonella. This is not a criticism and could be asked of any given study, but some form of explanation would benefit the manuscript.

Line 99: The choice of four clones is not clear at all to me. Which four clones? How do they relate to numbered descriptions in the previous results section?

Line 123; The point of mutations arising as a result of media adaptation could easily be resolved by sequencing some of the control populations evolved in the absence of streptomycin. Indeed I think this is a very important negative control that has been committed, and it is important to examine which mutations arise as a simple process of lab evolution. It seems unlikely given the intimate link to hypermutation but I think this should be done

Line 345: I think the authors need to be careful with their statement of significance here. All of these mutations only occurred in a hypermutator background and so this is a major caveat, especially given that recent publications have provided evidence that hypermutators are unlikely to persist long term in most given environments. Having said that I also think they miss another significance of their study in the discussion, namely the potential impact these findings could have on in silico AMR predictions

Line 423: The sequencing description is totally inadequate. The analysis done is mapping not assembly based. There is also no mention of mapping parameters employed, SNO calling parameters and cutoffs etc. as such the analysis is not reproducible. However far more distressing is the lack of provision of all raw sequence data. This is quite frankly wholly unacceptable

Minor Issues:
Line 68: Close brackets at beginning of line

Line 114: This is merely my opinion and a suggestion rather than a complaint, but i feel maybe some graphical representation of the mutations would be of benefit to the reader

Line 182. What happens when aadA is mutated in a strain evolving resistance at high MIC levels?

Line 265: Could you do a series of fitness experiments with the stepwise combinations of constructed mutants?

Line 411: Selected concentrations is vague. Please give full details of exactly which cones and which plates selected colonies were taken from

Response to reviewers:

Reviewers' comments:

Reviewer #1 (Remarks to the Author):

1. The manuscript submitted to Nature Communications by Wistrand-Yuen et al. reports how high-level streptomycin resistance emerges in response to low doses of streptomycin in salmonella enterica. In the abstract and in the introduction, authors report their main finding as "These results demonstrate how the strength of the selective pressure influences evolutionary trajectories and that even very weak selective pressures can cause evolution of high-level resistance.
" Although this is an important argument, the data provided by the authors do not provide much evidence to support that.

We make two statements in the manuscript: (i) the strength of the selective pressure influences evolutionary trajectories and (ii) even very weak selective pressures can cause evolution of high-level resistance.
The reviewer's claim that we do not provide support for those statements puzzles us since we would argue that they both follow directly from the experiments.

Regarding the first statement we think that this follows from the experimental data. We assume that what the reviewer refers to here and has a problem with is the point raised under point 5 below—"that it is not a fair comparison since the strong selection was done on plates after 24 hours of growth and the weak selection was in liquid culture after serial passage". This is a fair point and to further substantiate our claim we have performed a serial passage in liquid culture at high streptomycin levels. As observed for the plate selection the only resistance conferring mutations found are rpsL mutations. Thus, we would stand by our conclusion that the strength of the selective pressure influences the evolutionary trajectory. I.e. with a strong selection we get only rpsL mutants and with a weak selection we get other types of mutations (and no rpsL mutations) (see also response to points 5 and 6 below).

Regarding the second (and most important) statement our experimental data are absolutely clear---i.e. a selective pressure below MIC (1/4 of MIC, 1 ug/ml) can select for high-level resistance (>1024 ug/ml).

2. Besides, this finding is not novel and has been previously reported by others.

We respectfully disagree and it would certainly have been useful if the reviewer had provided a reference(s) to that claim. The main point of the manuscript is that very weak selective pressures <MIC) can select for high-level resistance by a combination of many mutations of small effect (note that the extent of epistasis between the 5 studied mutations is stronger than,

to our knowledge, has ever been reported. Thus, 4 out of 5 mutations hardly increased MIC by themselves but in combination they gave very high levels of resistance). To our knowledge this has not been observed before. In addition, we provide a mechanistic explanation for how these mutations confer their effect (again this is typically not done in the typical evolution experiments performed in this field).

I have several comments about the manuscript and unfortunately I do not think that the manuscript is suitable for publication at this stage.

The introduction and the abstract emphasize the importance of selection in low doses of antibiotics. I agree with the authors about the importance of this issue. Indeed, regardless of how antibiotics are utilized, there will always be residual (intact or degraded) antibiotic molecules in the environment and this may select for resistance.

3. However, the choice of antibiotic dosage for the laboratory evolution experiments for this is study is not justified. 25% of the MIC was used for the selection and authors report that this causes only ~3% growth rate reduction for the wild type ancestor strain. I do not understand how such a small change can be responsible for evolution of high levels of resistance and emergence of mutator strains (4 out of 10). Growth rate data for salmonella in different streptomycin concentrations are not provided therefore it is difficult for me to understand (predict) other changes caused by 1mg/l streptomycin.

The concentration chosen (1ug/ml) reduces growth by 3% (estimated by a competition experiment) and as we show this concentration is sufficient to select for high-level resistance (>1024 ug/ml). This concentration was chosen to represent a low concentration but still high enough to provide some selection. We have clarified the choice of antibiotic concentration (page 4, lines 22-25, page 5, line 1).

The finding that this low drug concentration can select for high-level resistance is indeed unexpected (and of course the main reason for why this study is interesting). However, the whole point of our modeling was to show how this can occur during 900 generations of evolution. As described, it only works if a mutator allele appears early in the experiment (which we also observe). The fact that mutators are enriched during adaptive evolution (especially when it involves sequential selection for several mutations, as occurred in our experiments) is well described in the literature (both experimentally and theoretically, see for example PLoS Comput Biol. 2010, 6:e1000710; Res Microbiol. 2001,152:11.; Genetics. 1999, 152:485; Microbiology. 2006, 152:67; Nature. 1997, 387:700; J. Bacteriol. 179, 417–422 (1997)) and is actually expected. We have further clarified that second-order selection of mutators is well established in previous studies (page 12, line 16-18).

4. Growth rate is not the only phenotype that should be monitored for fitness. The lag time or the yield can change as well. For example, mutations that reduce

the lag time in low dose antibiotics will certainly be selected. Besides, since fitness measurements were based on OD600 reads, corresponding cfu/ml counts could be useful to understand the level of selection by the drug dose used.

The reviewer is correct that selection can occur at many levels in a serial passage experiment. We have not distinguished between these effects since lag time and yield effects in the expected range (a few %) are very difficult to measure and not really relevant for the claims made in the manuscript. Furthermore, the 3% reduction in growth rate is not based on exponential growth rate measured with OD600, but on our previous work (Gullberg et al. 2011), where we observed that in a competition experiment, a 1 µg/mL concentration of streptomycin confers a 3% reduction in fitness. The manuscript has now been updated to clearly state this (page 4, lines 22-25; page 5, line 1).

5. One of the major claims of this work is the existence of rpsL mutations in only strong selection. I have read the relevant parts of the paper several times to clarify and I might still be missing something. I apologize in advance if this is due to my lack of understanding. According to the materials and methods section, the assay for strong selection was done using 24 hours of growth in 200mg/l streptomycin on solid agar, whereas the weak selection was done in small volumes of well shaken liquid media for 900 generations. If this is correct, this is not a fair comparison to begin with. It is known that resistant colonies selected on solid agar will not have multiple mutations. Typically, one mutation will be enough to survive. However, in liquid media, several sub-populations will compete and multiple mutations will emerge. This comparison is not fair since the drug concentration is not the only variable.

This is a good point. We have repeated the high-level selection by serial passage in liquid culture and whole genome sequenced 5 cultures. Also, under these conditions we see only rpsL mutations and none of the mutations found at low drug concentration (see also point 1).

To summarize, we have whole genome sequenced 11 isolates selected at high strep (5 obtained by serial passage in liquid culture and 6 obtained after plating) and find only rpsL mutations and no other resistance mutations. We also whole genome sequenced 6 isolates selected at sub-MIC and in those we find no rpsL mutations but only other resistance mutations (e.g. gidB, nuoG, cyoB, znuA, trkH).
Thus, our claim that different mutations are selected at high and low antibiotic level is solid. These new data have been added (page 4, lines 9-16).

6. My understanding is that only 4 (highly resistant) clones from weakly selected populations were sequenced by whole genome sequencing. I honestly could not understand whether clones selected in high doses were whole genome sequenced. If I am not wrong, only the rpsL gene was sequenced by Sanger sequencing. Therefore, it is not possible to rule out the existence of other mutations in strongly selected clones.

We have now whole genome sequenced 5 populations and 6 clones selected by serial passage at high streptomycin concentrations in liquid culture and agar plates, respectively. The only resistance conferring mutations found in those strains are rpsL mutations, again reinforcing the fact that the mutation selected at high and low concentrations are different. Thus, under high concentrations we always find rpsL mutations and during weak selection we find other types of mutations (but no rpsL mutation). This new sequencing data is now included (page 4, lines 9-16).

4. Authors chose 4 most resistant clones and whole genome sequenced them. I certainly understand that there might be financial constraints limiting the number of sequenced clones. However, I am a bit concerned that all of the sequenced strains turned out to be mutators. The remaining resistant clones may or may not be mutators and their genotypes are not known if I am not missing something. It was not possible to understand this from table S1 and the text. (i) they might have mutations in rpsL; (ii) they might not be mutators; (iii) they might have only single mutations. Therefore, the conclusions about the genetic pathways and mechanisms are not well supported by the sequencing data.

We chose to focus on the 4 strains with the highest resistance level (which all turned out to be mutators). We have whole genome sequenced two additional clones isolated on 96 µg/ml streptomycin from lineages with lower streptomycin resistance as well and the strains have been added to Table 1 (strains DA23877, 23879) and the sequence data is now included in Table S1. As can be seen they are not mutators but they have multiple mutations. Neither of them have mutations in rpsL, but both have likely loss-of-function mutations in gidB. We did not isolate any clones from the remaining 14 lineages, since they did not reach higher levels of streptomycin resistance within the 900 generations of evolution.

5. Examining the effects of five mutations in all possible combinations and examining the effect of aadA regulation is certainly very useful for everyone in the field. However, it does not add much to the main claims of the paper.

We respectfully disagree. A major point of the paper was to mechanistically explain how these different mutations (gidB, nuoG, cyoB, znuA and trkH) confer the increase in resistance.

6. The epistasis calculations can be done more systematically. The combined epistasis term is shown in figure 2. It clearly suggests that there is epistasis but it can be more informative if epistatic terms were calculated depending on their orders (1st order, 2nd order, 3rd order, 4th order). One can use the formalism described by Poelwijk et al. (Poelwijk FJ, PLoS Comput Biol 12(6): e1004771.). https://doi.org/10.1371/journal.pcbi.1004771. Also, using a log scale on the y axis of fig 2 might be helpful.

Thanks, this is a good suggestion. We have now calculated the higher order epistatic effects of the different resistance mutations through Walsh-

Hadamard transform decomposition of the fitness landscape as described by Poelwijk et al., with the full epistasis dataset presented in new Table S2. However, we disagree with the suggestions to change the y axis to log scale since the starting point in bar charts can not be set to 0. Depending on what starting point is chosen it will distort the bar graph possibly causing a misleading representation of the proportions. At the same time, we believe the data is best represented as a bar graph on a linear scale rather than a scatter plot on a log scale to visualize the amount of epistasis observed in the quintuple mutant.

7. Fig.4. Reduction in the intracellular uptake of streptomycin does not seem to be large enough to explain elevated streptomycin resistance. Since all of these strains were already phenotyped for resistance, it should be straightforward to calculate the correlation.

Yes, we agree that there was no big change in streptomycin uptake. Thus, the triple mutant (DA40378-trkH, cyoB, nuoG, see Table 2) had only a 30% reduction in uptake but a 4-fold increase in MIC. However, there is no a priori reason to expect that the relationship between streptomycin uptake and resistance level should correlate in a linear and proportional way. Thus, a small effect on uptake could have a large effect on resistance. We have added a comment about this in the manuscript (page 10, lines 12-15). Additionally, we likely underestimate the reduction in uptake since we can not estimate the background signal of extracellular bound streptomycin. If a substantial amount of streptomycin is bound extracellularly this would lower the proportion that can be seen by a reduction of intracellular streptomycin. We have added a comment about this (page 10, lines 15-17).

Reviewer #2 (Remarks to the Author):

Review Wistrand-Yuen et al- Nature Communications 2017

This is a very interesting and well written manuscript demonstrating how selection strength influence the evolutionary trajectories of antibiotic resistance. Whereas this has been indicated in previous work, this paper the first to address this mechanistically through elegant reconstructions of combinations of all mutations predicted to play a role in antibiotic (streptomycin) resistance.

The authors present a "fitness landscape" of all possible combinations of five mutations predicted to play a role in evolution of streptomycin resistance. Interestingly, whereas only one of these mutations significantly elevated resistance alone, the combination of all five mutations resulted in high level streptomycin resistance through the combined effects of three different mechanisms (target modification, reduced uptake and induction of a chromosomal aminoglycoside modifying enzyme). The authors present compelling evidence of strong positive epistasis between individual resistance mutations of low effect. The emergence of several mutations with reduced

fitness (without immediate benefit) during 900 generations of experimental evolution is rationalized by a mathematical model.

This is solid work with properly controlled experiments and it is of great interest to a broad scientific audience. The work increases our understanding of how low concentrations of antibiotics may select for high level resistance (i.e. clinically relevant) through a remarkable genetic repertoire of atypical resistance mutations. This paper will, if my points below are addressed adequately, significantly influence thinking in the field.

Critical points:

Whereas I do not doubt that resistance increases stepwise with combinations of several of these mutations Table 2 displays several intermediate MIC values Please include a description of how the MICs were determined with respect to
a) Number of experiments
b) If MICs were modes or averages of several repeated experiments.

Several intermediate MICs are reported as values below 2- fold changes. This is arguably problematic since clinical laboratories often require 4- fold changes in both directions in order to report significant changes in susceptibilities. However, if the values are based on multiple biological replicates this point can be ignored.

We understand the concern, however, the Etest scale has higher resolution than the 2-fold scale used clinically, and the MIC values presented in the manuscript are the median values of 2 to 5 Etests from biological replicates. This has now been clarified in the manuscript (Table 1 text lines 3-4 and Table 2 text lines 4-5).

Please go over Table 2 and correct txt, or table- several inconsistencies:

2. P 7, line 3: gidB mutation MIC = 48 mg/L – Table 2 reports 32 mg/L.

This has been corrected.

3. P 7, line 4: znuA is reported to result in 2- fold change- listed in Table 2 as 1,5 fold change- significant?

This has been corrected.

4. P7, line 11: gidB + both respiratory chain mutations is listed as MIC of 256 mg/L, and not 192 in Table 2.

This has been corrected.

5. P7, line 13: quadruple mutant MIC og 192- right?

This has been corrected.

6. P8, line 16: Table 2 lists 7 cases, and Table 1 a single case, where no reductions in MIC were reported following deletion of relA and spoT

This has been corrected.

7. P13, line 24: 50-100 fold decrease: this does not reflect the numbers reported in Tables 1 and 2- please correct.

This has been corrected.

8. P15 and 16 lines 24 and 1, respectively. Technically this may be true- I believe the authors mean downregulation of aadA- right?

Yes, we mean downregulation of aadA, this has been corrected.

9. The astute reader will observe that the combination of gidB and cyoB alone (Strain DA23872) gave a MIC of 512 mg/L. In table 1, the reconstruction show that the presence of these 2 mutations gave an MIC of 96 mg/L. The authors might consider a short elaboration of the possibility that many more mutations of small effects may contribute to high level resistance.

This is a good point. The reconstructed strains are all based on the specific mutations found in the mutant with the highest MIC, DA23869. We have not tried to reconstruct the specific mutations of DA23872, but we agree that many more mutations of small effect may contribute to the resistance. This specific strain has other potential resistance mutations, such as znuB (Trp73*), a gene that is part of the same zinc transport system as znuA, as well as mutations that might affect membrane potential such as fdoG, a component of formate dehydrogenase, and hemF, an enzyme in the heme biosynthesis pathway. We have commented on the possibility of additional mutations contributing to the resistance phenotype (page 12, lines 1-3).

Other points:

10. Lack of proper fitness estimates is well explained in the discussion. However, the Lenski lab (and others) have reported in a series of papers competitions with mutator E. coli evolved strains and populations. I assume that the problem here is the small-fitness effects of individual mutations and that longer time series needed to accurately measure these are hampered by deviations from linearity in the competition experiments and that shorter competition experiments did not result in sufficient resolution- could this be worth explicitly stating?

Yes, this is correct—since the strains are mutators they will rapidly pick up media adaptation mutations that prevent accurate measurements of small fitness differences (<1%). We have clarified this further (page 13, lines 3-7).

11. Model: I find it slightly under explained- whereas it is mentioned in the discussion that the rather narrow bottlenecks are included (Ne component) I

believe it would benefit readers that hums rather than solves equations to explicitly explain this.

We have added some further clarification of the model (page 10, lines 19-24).

- The manuscript would also benefit from a parameter-table.

We think that it is sufficient to provide the chosen parameter values in the text and figure legends.

- Table 3 needs to be better explained- it is difficult to understand (at least the version I got for review).

This table is now better explained in the legend.

- Figure 5 would also benefit from a revision where Y- axes are clearly explained and the Fig txt should be fully explanatory

Yes, we agree. Figure 5 has been revised with clearly explained Y-axes, and the graphs have been better explained in the legend (page 37, lines 3-5).

12. Concentration of sucrose used is missing in material and methods,

This has been corrected.

13. Concentrations of streptomycin on plating assays.

This has been added.

14. Supp Table 2: Explain DIP (deletion insertion polymorphisms?)
15. * on Q169 mutation (Table 2) (stop codon?)

Yes, DIPs are deletions, insertions and other polymorphisms, and the symbol "*" represents a stop codon. We have specified the mutation types better now, so that mutation types are listed as either single-nucleotide variants (SNVs), two or more SNVs in succession (MNVs), deletions or insertions. This has been clarified in the text and in the table legend of Table S1.

Reviewer #3 (Remarks to the Author):

The authors present a comprehensive analysis of the evolutionary landscape of Salmonella Typhimurium when exposed to sub-MIC levels of streptomycin. The level of molecular biology and mechanistic research undertaken is somewhat of a tour-de-force, and the findings represent novel and important conclusions for all working on AMR.

However I have a few points I would like to raise with the authors in the interest of possibly strengthening the presented manuscript.

Major issues:
An obvious question that immediately came to my mind is why use Salmonella. This is not a criticism and could be asked of any given study, but some form of explanation would benefit the manuscript.

Salmonella is a very common model organism for bacterial genetics, and much of the previous work in the field of antibiotic resistance has been done in Salmonella. We have added a comment about this (page 3, lines 20-21).

Line 99: The choice of four clones is not clear at all to me. Which four clones? How do they relate to numbered descriptions in the previous results section?

The four clones were chosen because they had the highest resistance. This has been clarified in the text (page 5, lines 20-21).

Line 123; The point of mutations arising as a result of media adaptation could easily be resolved by sequencing some of the control populations evolved in the absence of streptomycin. Indeed I think this is a very important negative control that has been committed, and it is important to examine which mutations arise as a simple process of lab evolution. It seems unlikely given the intimate link to hypermutation but I think this should be done.

The control populations evolved in MH without streptomycin have already been whole genome sequenced and published, and mutations in common between these control populations and our mutants as well as the reference to that paper has been added to the manuscript (page 8, lines 11-13).

Line 345: I think the authors need to be careful with their statement of significance here. All of these mutations only occurred in a hypermutator background and so this is a major caveat, especially given that recent publications have provided evidence that hypermutators are unlikely to persist long term in most given environments. Having said that I also think they miss another significance of their study in the discussion, namely the potential impact these findings could have on in silico AMR predictions

Good point. We have added a comment regarding in silico predictions in the Discussion (page 17, lines 18-20).

Line 423: The sequencing description is totally inadequate. The analysis done is mapping not assembly based. There is also no mention of mapping parameters employed, SNO calling parameters and cutoffs etc. as such the analysis is not reproducible. However far more distressing is the lack of provision of all raw sequence data. This is quite frankly wholly unacceptable

We agree completely and apologize for the poor description. The sequencing description has been rewritten, and the raw sequence data has been provided (page 19, lines 16-25; page 20, lines 1-25; page 21, lines 1-5, SRA accession no SRP133288).

Minor Issues:
Line 68: Close brackets at beginning of line.

This has been corrected.

Line 114: This is merely my opinion and a suggestion rather than a complaint, but i feel maybe some graphical representation of the mutations would be of benefit to the reader.

We respectfully disagree and do not think that it would add much to the manuscript. It would become a very messy figure since there are so many mutations.

Line 182. What happens when aadA is mutated in a strain evolving resistance at high MIC levels?

Since resistance evolution of Salmonella at high levels of streptomycin generally selects rpsL mutants, we believe that this would also be the case for a strain lacking a functional aadA, since this resistance mechanism is aadA independent. This can be observed in E. coli, an organism that is related to Salmonella, but that lacks the aadA gene.

Line 265: Could you do a series of fitness experiments with the stepwise combinations of constructed mutants?

This could be done, and the fitness of the reconstructed strains might give an indication towards the possible order of appearance of the mutations. However, there are several additional mutations in the isolated mutants that could increase fitness without increasing the resistance level, such as media adaptation or mutations that compensate for the cost of the resistance mutations, so it would have limited value for this study.

Line 411: Selected concentrations is vague. Please give full details of exactly which cones and which plates selected colonies were taken from.

The different concentrations have now been specified, and the selected colonies have been described in more detail. All isolated clones have now been listed in new Table S5 (page 19, lines 10-12).

REVIEWERS' COMMENTS:

Reviewer #2 (Remarks to the Author):

I have now read the revised version of "Evolution of high-level resistance during low-level antibiotic exposure" submitted by Wistrand-Yuen and co-workers.

The authors have in my opinion more than adequately responded to major and minor issues concerns raised in the first round of reviews. The revised version is much improved and will be interesting for a broad range readers with interests in the evolution and spread of antimicrobial drug resistance.

Reviewer #3 (Remarks to the Author):

In the interests of fairness I have reviewed the revised manuscript based solely on responses to my original comments and not those of the other reviewers.

As such I thank the authors for their considered responses to my comments. In particular I appreciate the efforts to make the bioinformatics analysis reproducible by providing full details of analysis parameters and a link to the raw data.

I have no further comments to make on the manuscript, and I hope to look forward to seeing how the work is received by the community
