# Supplementary material for: Evolution of high-level resistance during low-level antibiotic exposure
Source: Nat Commun. 2018 Apr 23;9:1599. doi: 10.1038/s41467-018-04059-1 (PMC5913237; doi:10.1038/s41467-018-04059-1)
Supplement: Supplementary file 2 — Peer Review File [file 41467_2018_4059_MOESM2_ESM.pdf]

Supplementary Figures 1-2 and Supplementary Tables 1-8 for:

Evolution of high-level resistance during low-level antibiotic exposure

Wistrand-Yuen et al.

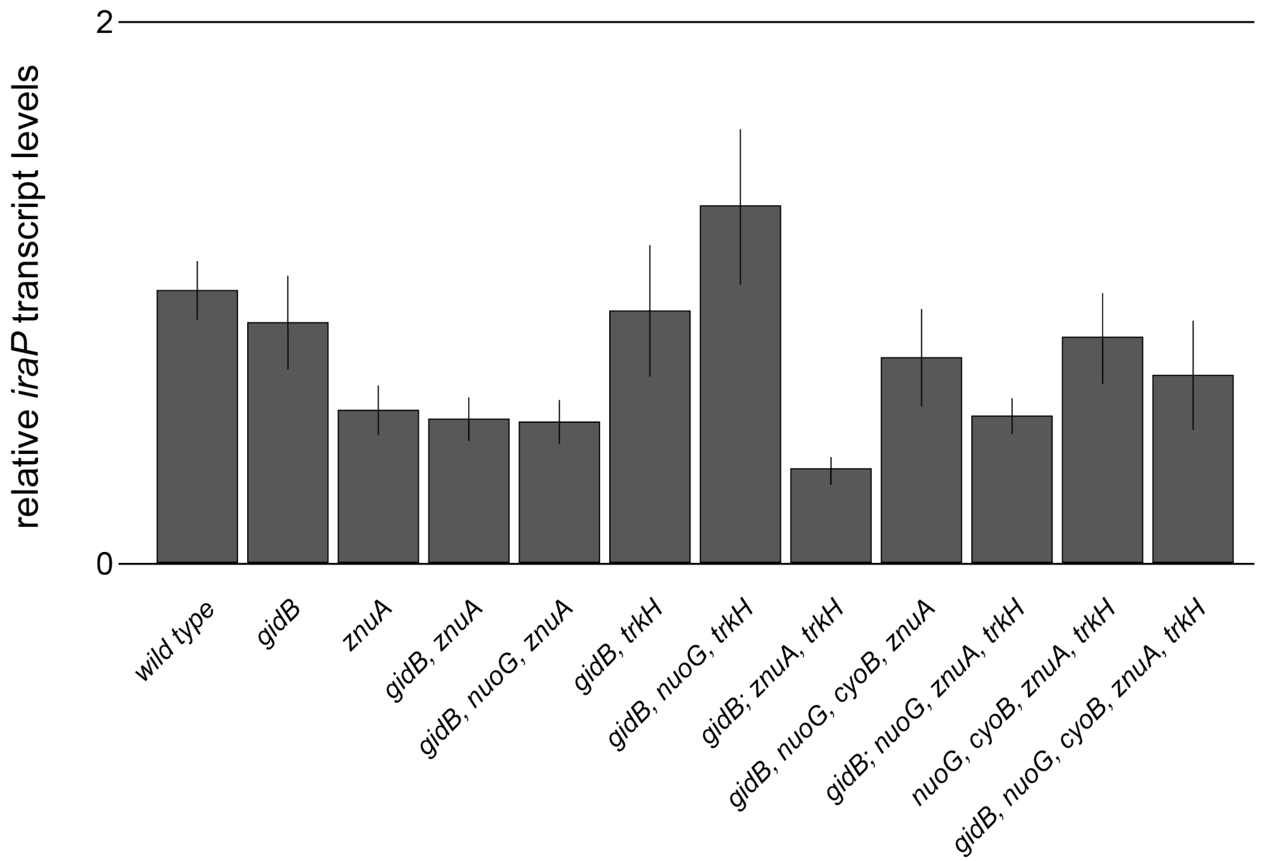

Supplementary Figure 1. Relative transcript levels of *iraP* in the wild type and selected mutants. No significant upregulation was observed in any of the samples. The values represent averages of 2 biological and 3 technical replicates. Error bars represent the standard deviation.

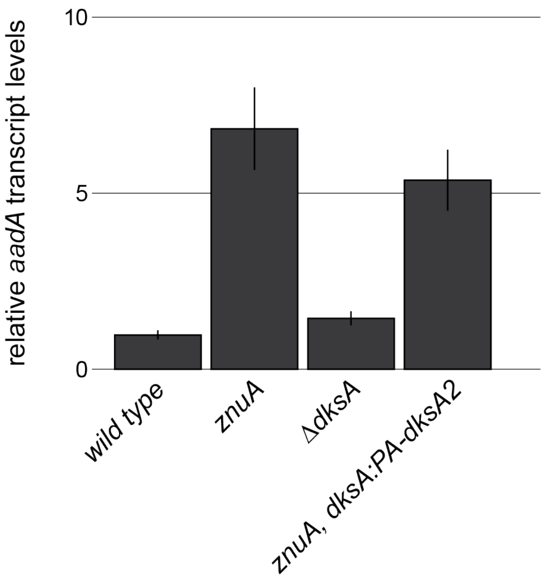

Supplementary Figure 2. Relative transcript levels of *aadA*. Expression of *aadA* was measured in wild type, reconstructed *znuA*-mutant, *dksA* knockout and a *znuA* mutant with a replacement of the native *dksA* gene with a zinc independent homologue from *Pseudomonas aeruginosa* (*PA-dksA2*). Error bars represent the standard deviation.

Supplementary Table 1. All mutations in the isolated mutants compared to wild type. Mutation types are single-nucleotide variants (SNVs), two or more SNVs in succession (MNVs), deletions and insertions. The symbol "*" represents a stop codon, while "fs" represents a frame shift mutation.

Strain	Position	Type	Annotation	Amino acid change
**DA23868**	85237	SNV	CDS: caiB	Val54Ala
**DA23868**	160039	SNV	CDS: secA	Pro451Ser
**DA23868**	225305	SNV	CDS: fhuA	Thr523Ala
**DA23868**	245972	Insertion		
**DA23868**	255731	SNV	CDS: tsf	Arg151Cys
**DA23868**	297812	Insertion	CDS: putative drug efflux protein	Val302fs
**DA23868**	302541	SNV	CDS: yafS	Pro130Leu
**DA23868**	302560	SNV	CDS: yafS	
**DA23868**	335622	SNV	CDS: putative RHS-like protein	Ala1024Val
**DA23868**	364624	Deletion	CDS: crl	Phe35fs
**DA23868**	370376	SNV		
**DA23868**	470999	Insertion		
**DA23868**	547591	SNV	CDS: hemH	
**DA23868**	680367	Deletion	CDS: rna	Asn145fs
**DA23868**	682157	SNV	CDS: citT	Tyr72Cys
**DA23868**	723009	SNV	CDS: hscC	Asp403Asn
**DA23868**	985689	SNV	CDS: hypothetical protein, CDS: hypothetical protein	His58Arg, Val150Ala
**DA23868**	1006741	SNV	CDS: putative nucleoside-diphosphate-sugar epimerase	Tyr264His
**DA23868**	1015778	Insertion		
**DA23868**	1133653	Insertion	CDS: host specificity protein J	Ala294fs
**DA23868**	1164914	SNV	CDS: yccS	Val307Ala
**DA23868**	1220293	SNV	CDS: putative inner membrane protein	Ala282Val
**DA23868**	1258842	Insertion	CDS: flgC	Val64fs
**DA23868**	1271172	SNV	CDS: rne	Tyr126His
**DA23868**	1343532	SNV	CDS: putative ABC-type transport system ATPase component	
**DA23868**	1346537	SNV		
**DA23868**	1357187	SNV	CDS: yeaL	Leu71Pro
**DA23868**	1363410	Deletion	Gene: yeaG	
**DA23868**	1375969	Deletion	CDS: selD	Arg126fs

**DA23868**	1527411	SNV	CDS: tppB	Trp179*
**DA23868**	1582873	SNV	CDS: rspA	His211Arg
**DA23868**	1593976	Deletion		
**DA23868**	1599457	SNV	CDS: ydeA	
**DA23868**	1610520	SNV	CDS: putative hydrogenase protein	Asp55Asn
**DA23868**	1618287	SNV	CDS: putative zinc-binding dehydrogenase	Trp18*
**DA23868**	1637147	SNV	CDS: putative glycosyl hydrolase	Val328Ala
**DA23868**	1639479	SNV	CDS: putative glycosyl hydrolase	
**DA23868**	1642462	Insertion	CDS: putative alpha amylase	Ala12fs
**DA23868**	1659045	SNV		
**DA23868**	1668866	SNV	CDS: nhoA	Glu117Gly
**DA23868**	1802376	SNV	CDS: yciM	Thr102Ala
**DA23868**	1845156	SNV	CDS: adhE	
**DA23868**	1862985	SNV	CDS: narK	Thr318Ala
**DA23868**	1870891	SNV	CDS: kdsA	
**DA23868**	1981602	SNV	CDS: yebK	Ala41Thr
**DA23868**	1984419	SNV	CDS: msbB	
**DA23868**	2064558	SNV	CDS: fliN	Asp9Asn
**DA23868**	2071971	SNV	CDS: yedI	Ala274Val
**DA23868**	2075768	SNV	RBS: RBS,	
**DA23868**	2092313	SNV	CDS: amn	
**DA23868**	2101006	SNV	CDS: cbiP	
**DA23868**	2149558	Insertion		
**DA23868**	2190052	Deletion	CDS: wcaI	Ile371fs
**DA23868**	2197408	SNV	CDS: wcaC	
**DA23868**	2436280	SNV	CDS: nuoE	Val69Ala
**DA23868**	2469205	SNV	CDS: putative cytoplasmic protein	Ala183Thr
**DA23868**	2690148	SNV	CDS: putative inner membrane protein	
**DA23868**	2712523	Insertion	CDS: yfhD	Val44fs
**DA23868**	2739118	Insertion	CDS: host specificity protein-J-like	Leu561fs
**DA23868**	2750091	SNV	CDS: minor capsid protein FII	
**DA23868**	2783659	SNV	CDS: srmB	
**DA23868**	2836458	SNV	CDS: STM2689	Val2959Ile
**DA23868**	2841809	SNV	CDS: putative ABC transporter transmembrane region	
**DA23868**	2847103	Insertion	CDS: phage tail-like protein	Val669fs
**DA23868**	2858360	SNV	CDS: phage tail-like protein	Gly52Ser
**DA23868**	2921898	SNV	CDS: iroD	

**DA23868**	2930399	Deletion	CDS: mig-14	Lys269fs
**DA23868**	2966261	SNV		
**DA23868**	2970562	SNV	tRNA: tRNA-Ser	
**DA23868**	2984651	SNV	CDS: ygaA	
**DA23868**	2996059	SNV	CDS: hycD	Ile164Thr
**DA23868**	3052487	SNV	CDS: mutS	Leu734Pro
**DA23868**	3060226	SNV	CDS: ygbK	Arg165Cys
**DA23868**	3083757	SNV	CDS: putative cytoplasmic protein	Phe94Leu
**DA23868**	3089591	SNV	CDS: cysI	Thr377Ala
**DA23868**	3101011	SNV	CDS: mazG	
**DA23868**	3111480	SNV	CDS: gudD	
**DA23868**	3129408	SNV	CDS: fucI	
**DA23868**	3132832	SNV	CDS: ygdE	
**DA23868**	3150051	SNV	CDS: ptr	Arg831Cys
**DA23868**	3161257	Insertion	CDS: ptsP	Gln303fs
**DA23868**	3186121	SNV	CDS: hypothetical protein	Arg133Cys
**DA23868**	3392258	Deletion		
**DA23868**	3397070	SNV	CDS: yqjD	Ala89Thr
**DA23868**	3418947	SNV	CDS: garD	Val91Ile
**DA23868**	3445076	SNV	CDS: mtr	Arg322Cys
**DA23868**	3532372	SNV	CDS: yhcQ	Thr181Met
**DA23868**	3539635	SNV	CDS: yhdP	
**DA23868**	3541535	SNV	CDS: maf	Thr125Ala
**DA23868**	3548156	Insertion	CDS: putative sulfite oxidase subunit YedY	Met20fs
**DA23868**	3563809	SNV	CDS: acrF	Gly510Asp
**DA23868**	3587650	SNV	CDS: rplO	
**DA23868**	3592121	SNV	CDS: rplP	Asp106Gly
**DA23868**	3676829	Insertion		
**DA23868**	3681105	SNV	CDS: rtcA	
**DA23868**	3681839	SNV	CDS: rtcB	Ala240Thr
**DA23868**	3711068	SNV	CDS: gntU	
**DA23868**	3808106	SNV		
**DA23868**	3945220	SNV	CDS: yicI	Tyr551Cys
**DA23868**	3997538	SNV	CDS: ilvB	
**DA23868**	4049508	SNV	CDS: trmE	Asp440Asn
**DA23868**	4063764	SNV	CDS: pstS	
**DA23868**	4064947	SNV	CDS: putative phosphotransferase system	Ala338Val

**DA23868**	4081716	SNV	CDS: gidB	Gln22*
**DA23868**	4086386	SNV	CDS: yieM	Ala450Val
**DA23868**	4131118	SNV	CDS: rffG	Gln222*
**DA23868**	4179218	SNV		
**DA23868**	4266460	SNV	CDS: yiiM	
**DA23868**	4290951	SNV	CDS: yiiR	Met112Val
**DA23868**	4320353	SNV	CDS: katG	
**DA23868**	4403441	SNV	CDS: aceA	Thr332Ala
**DA23868**	4441487	SNV	CDS: yjbF	Ala60Val
**DA23868**	4448384	SNV	CDS: malE	Phe305Ser
**DA23868**	4553316	SNV	CDS: putative anaerobic dimethylsulfoxide reductase subunit B	Pro72Leu
**DA23868**	4571807	SNV	CDS: fxsA	Pro103Ser
**DA23868**	4634053	SNV	CDS: cycA	Val143Ala
**DA23868**	4643962	Insertion		
**DA23868**	4696458	Insertion	Gene: treB	
**DA23868**	4720045	SNV	CDS: pepA	
**DA23868**	4739921	Insertion	CDS: putative cytoplasmic protein	Glu79fs
**DA23868**	4739981	SNV	CDS: putative cytoplasmic protein	Arg57His
**DA23868**	4784944	SNV	CDS: putative cytoplasmic protein	Tyr81His
**DA23868**	4842823	SNV	CDS: creA	Ala99Val
**DA23869**	391	SNV	CDS: thrA	Arg19Cys
**DA23869**	76853	SNV	CDS: carA	Leu325Pro
**DA23869**	81198	SNV	CDS: caiE	Val140Ala
**DA23869**	154309	Insertion	CDS: ftsQ	Glu262fs
**DA23869**	164202	SNV	CDS: guaC	
**DA23869**	185131	SNV	CDS: yacH	Ala113Val
**DA23869**	230358	Insertion		
**DA23869**	234939	SNV	CDS: stfE	Gly64Arg
**DA23869**	245174	SNV	CDS: htrA	Thr228Ala
**DA23869**	248205	SNV	CDS: putative inner membrane protein	Thr85Met
**DA23869**	252048	SNV	CDS: glnD	Ala257Thr
**DA23869**	258138	SNV	CDS: dxr	Met98Val
**DA23869**	302704	Insertion	CDS: yafS	Arg187fs
**DA23869**	316973	SNV	CDS: putative cytoplasmic protein	Val27Ala
**DA23869**	321982	SNV	CDS: hypothetical protein	Pro291Ser
**DA23869**	326320	SNV	CDS: putative inner membrane protein	Asn820Ser
**DA23869**	342584	SNV	CDS: safA	

**DA23869**	350623	SNV		
**DA23869**	352334	SNV	CDS: yafV	
**DA23869**	357677	SNV		
**DA23869**	368821	SNV	tRNA: thrW	
**DA23869**	443225	SNV	CDS: rdgC	Val206Ala
**DA23869**	496334	SNV	CDS: cyoB	Gly283Asp
**DA23869**	496381	SNV	CDS: cyoB	
**DA23869**	505578	SNV	CDS: lon	
**DA23869**	513676	Insertion	CDS: ybaE	Ser153fs
**DA23869**	546507	SNV	CDS: adk	Thr155Ile
**DA23869**	622700	SNV		
**DA23869**	774925	SNV	CDS: phrB	Pro49Ser
**DA23869**	806753	SNV	CDS: sucC	
**DA23869**	904789	SNV	CDS: ybiS	Ala11Val
**DA23869**	912823	SNV	CDS: pflF	Trp320*
**DA23869**	1110307	SNV	CDS: hypothetical protein	
**DA23869**	1110316	SNV	CDS: hypothetical protein	
**DA23869**	1110325	SNV	CDS: hypothetical protein	
**DA23869**	1162292	Insertion		
**DA23869**	1224740	Insertion	tRNA: serX	
**DA23869**	1232990	Deletion	RBS: RBS	
**DA23869**	1254229	SNV	CDS: mviM	
**DA23869**	1265494	SNV	CDS: flgK	Asn13Asp
**DA23869**	1363410	Deletion	Gene: yeaG	
**DA23869**	1405271	SNV	CDS: pfkB	
**DA23869**	1487338	Deletion	Misc. feature: SPI-2, CDS: sseE	Ala100fs
**DA23869**	1597469	SNV	CDS: marA	Met1?
**DA23869**	1598486	SNV	CDS: marC	
**DA23869**	1611940	SNV	CDS: putative Ni/Fe hydrogenase 1 b-type cytochrome subunit	Tyr34His
**DA23869**	1633128	SNV	CDS: putative transcriptional regulator	
**DA23869**	1650197	SNV	CDS: fdnH	Ser76Pro
**DA23869**	1653692	SNV		
**DA23869**	1781866	SNV	CDS: pspA	Ala182Val
**DA23869**	1788214	SNV	CDS: sapF	
**DA23869**	1885035	SNV	CDS: hydrogenase-1 small subunit	
**DA23869**	1941094	Deletion		
**DA23869**	1987304	Insertion	CDS: znuA	Ser19fs

**DA23869**	1992784	SNV	CDS: yebB	Ser54Gly
**DA23869**	1999723	SNV	CDS: yecO	
**DA23869**	2016562	SNV		
**DA23869**	2029377	SNV	RBS: RBS	
**DA23869**	2061641	SNV	CDS: fliJ	Gln115*
**DA23869**	2085229	Insertion		
**DA23869**	2095178	SNV	CDS: yeeO	Gln219*
**DA23869**	2178644	SNV		
**DA23869**	2295767	SNV	CDS: putative regulatory protein	Ala195Thr
**DA23869**	2433830	SNV	CDS: nuoG	Asp264Gly
**DA23869**	2446041	SNV	CDS: yfbT	
**DA23869**	2503789	SNV	CDS: vacJ	Glu244Lys
**DA23869**	2592702	SNV	CDS: narQ	Thr314Met
**DA23869**	2742856	SNV	CDS: phage tail component M-like protein	*110Trp
**DA23869**	2798241	Insertion	rRNA: rRNA	
**DA23869**	2816335	SNV	CDS: rimM	
**DA23869**	2821018	Insertion	CDS: putative cytoplasmic protein	Leu25fs
**DA23869**	2845190	SNV	CDS: late control-like protein	
**DA23869**	2912853	SNV	CDS: fljA	
**DA23869**	2940273	Insertion	CDS: gabD	Ala47fs
**DA23869**	3069492	SNV	CDS: truD	
**DA23869**	3079887	SNV	CDS: ygcH	Thr76Ala
**DA23869**	3079906	SNV	CDS: ygcH	
**DA23869**	3107032	SNV	CDS: barA	Val169Ala
**DA23869**	3143285	SNV	CDS: argA	Asp146Gly
**DA23869**	3147163	SNV	CDS: RecB	Val833Ala
**DA23869**	3169658	SNV	CDS: aas	
**DA23869**	3185444	SNV	CDS: putative cytoplasmic protein	Val192Ala
**DA23869**	3245214	SNV	CDS: putative mannitol dehydrogenase	Leu211Pro
**DA23869**	3257064	SNV	CDS: gshB	Met40Val
**DA23869**	3287613	SNV	CDS: putative oxidoreductase	
**DA23869**	3368021	SNV	CDS: ygiF	Gln97Arg
**DA23869**	3392258	Deletion		
**DA23869**	3415077	Insertion	Misc. RNA: rnpB	
**DA23869**	3482894	SNV	CDS: yrbK	Ala119Thr
**DA23869**	3552533	SNV	CDS: panF	Val87Ala
**DA23869**	3730830	SNV	CDS: livM	

**DA23869**	3794167	SNV	CDS: yhjL	Arg1083Cys
**DA23869**	3836547	SNV	CDS: cspA	
**DA23869**	3939879	SNV	CDS: gltS	Pro375Ser
**DA23869**	3965999	SNV	CDS: yicL	Thr111Ala
**DA23869**	3974022	SNV	CDS: putative transcriptional regulator	Thr435Ala
**DA23869**	3999035	SNV	CDS: integral membrane protein	Ala136Thr
**DA23869**	4003260	SNV	CDS: dsdX	
**DA23869**	4010699	SNV	CDS: yidQ	
**DA23869**	4028522	SNV	CDS: torS	Met698Ile
**DA23869**	4081275	SNV	CDS: gidB	Gln169*
**DA23869**	4096715	SNV	CDS: rbsR	Leu65Pro
**DA23869**	4158371	Insertion	CDS: yigG	Asp146fs
**DA23869**	4192743	SNV	CDS: trkH	Thr99Ala
**DA23869**	4300185	SNV	CDS: ftsN	Ala227Thr
**DA23869**	4302505	SNV	CDS: priA	Tyr611His
**DA23869**	4446742	SNV	CDS: malF	Gln412*
**DA23869**	4519496	SNV	CDS: nrfE	Thr296Ala
**DA23869**	4600831	Deletion	CDS: mutL	Trp121del
**DA23869**	4706682	SNV	CDS: ornithine carbamoyltransferase	Val124Ile
**DA23869**	4793345	SNV	CDS: putative transcriptional regulator	Glu833Gly
**DA23869**	4798813	Insertion	CDS: mdoB	Arg654fs
**DA23869**	4809418	Insertion	tRNA: leuV	
**DA23869**	4818547	SNV	CDS: yjjI	Arg469His
**DA23872**	50727	SNV	CDS: putative sodium galactoside symporter	
**DA23872**	84700	SNV	CDS: caiB	Tyr233Cys
**DA23872**	122305	SNV	CDS: araC	
**DA23872**	171432	SNV	CDS: putative cytoplasmic protein	Phe127Leu
**DA23872**	193565	SNV	CDS: putative transcriptional regulator	
**DA23872**	205743	SNV	CDS: stiC	Ser628Asn
**DA23872**	207172	SNV	CDS: stiC	Tyr152His
**DA23872**	233564	SNV	CDS: stfC	Ala754Val
**DA23872**	242024	SNV	CDS: btuF	Leu16Pro
**DA23872**	267043	SNV	CDS: lpxA	
**DA23872**	310051	Deletion	CDS: putative cytoplasmic protein	Thr261fs
**DA23872**	321801	Insertion	CDS: hypothetical protein	Ala232fs
**DA23872**	327522	SNV	CDS: putative inner membrane protein	Thr1221Ala
**DA23872**	347953	SNV		

**DA23872**	389590	SNV	CDS: putative inner membrane protein	Ser31Gly
**DA23872**	404378	SNV	CDS: mod	
**DA23872**	428383	SNV	CDS: yaiV	Gln66*
**DA23872**	446732	SNV	CDS: sbcC	Leu884Pro
**DA23872**	446962	SNV	CDS: sbcC	
**DA23872**	561004	SNV	CDS: ybbJ	Gly142Asp
**DA23872**	573832	SNV	CDS: ybbB	Thr318Ala
**DA23872**	625777	SNV	CDS: ybdG	Val370Ala
**DA23872**	673838	SNV	CDS: putative oxidoreductase protein	
**DA23872**	676897	SNV	CDS: putative hydrogenase protein	Met120Val
**DA23872**	681121	SNV	CDS: citT	
**DA23872**	685408	SNV	CDS: citE	Leu291Pro
**DA23872**	724743	Insertion	CDS: putative cytoplasmic protein	Leu263fs
**DA23872**	779010	SNV	CDS: ybgJ	
**DA23872**	796338	SNV	CDS: gltA	
**DA23872**	840871	SNV	CDS: galE	Val244Ile
**DA23872**	919408	Deletion	CDS: yliA	Asp437fs
**DA23872**	975401	SNV		
**DA23872**	1018676	Insertion	CDS: ybjY	Met24fs
**DA23872**	1070548	SNV	CDS: msbA	
**DA23872**	1099833	SNV		
**DA23872**	1215181	SNV		
**DA23872**	1224740	Insertion	tRNA: serX	
**DA23872**	1301502	SNV	CDS: mfd	Pro277Leu
**DA23872**	1556640	SNV	CDS: pntA	Arg318Cys
**DA23872**	1560683	SNV	CDS: ydgE	Gly31Ser
**DA23872**	1642275	SNV	CDS: putative alpha amylase	Val74Ala
**DA23872**	1732273	SNV	CDS: hrpA	Arg968Cys
**DA23872**	1788452	SNV	CDS: sapF	Ser100Pro
**DA23872**	1813959	SNV	CDS: sohB	
**DA23872**	1838108	SNV	CDS: oppC	Phe147Leu
**DA23872**	1862986	Insertion	CDS: narK	Thr318fs
**DA23872**	1879670	SNV	CDS: ychM	
**DA23872**	1988406	SNV	CDS: znuB	Trp73*
**DA23872**	2021974	SNV	CDS: flhD	Asp31Gly
**DA23872**	2026179	SNV	CDS: araH	Asp170Gly
**DA23872**	2210000	SNV	CDS: yegE	Val491Ala

**DA23872**	2444194	Deletion	CDS: yfbS	Val543fs
**DA23872**	2561611	SNV	CDS: hemF	Gly136Asp
**DA23872**	2615307	SNV	CDS: ppk	Arg124His
**DA23872**	2645744	SNV	CDS: ratA	Asp577Gly
**DA23872**	2646287	SNV	CDS: ratA	Asp396Gly
**DA23872**	2737238	SNV		
**DA23872**	2777392	SNV	CDS: rseC	Pro136Ser
**DA23872**	2823773	SNV	CDS: recN	Ser169Asn
**DA23872**	2824008	SNV	CDS: recN	
**DA23872**	2925343	SNV	CDS: iroN	
**DA23872**	2937830	SNV	RBS: RBS	
**DA23872**	3094368	SNV		
**DA23872**	3168903	SNV	CDS: aas	His379Arg
**DA23872**	3175670	Insertion	CDS: ygeA	Met10fs
**DA23872**	3263489	SNV	CDS: yggW	Ile123Thr
**DA23872**	3279213	SNV	CDS: putative transcriptional regulator	Thr122Ala
**DA23872**	3344168	SNV	CDS: parE	
**DA23872**	3359814	SNV	CDS: putative inner membrane protein	Ser9Asn
**DA23872**	3631572	Deletion	CDS: bigA	His802fs
**DA23872**	3678781	SNV	CDS: malT	Glu636*
**DA23872**	3683699	SNV	CDS: putative ribonucleoprotein related-protein	Asn319Ser
**DA23872**	3829179	SNV		
**DA23872**	3835753	Insertion		
**DA23872**	3909892	Insertion	CDS: rfaK	Gln265fs
**DA23872**	3978385	SNV	CDS: nepI	
**DA23872**	4068130	SNV	CDS: putative dipeptide/oligopeptide/nickel ABC-type transport system periplasmic component	
**DA23872**	4081556	SNV	CDS: gidB	Gly75Glu
**DA23872**	4244279	SNV	CDS: fdoG	Ala769Val
**DA23872**	4414847	SNV	CDS: yjbC	Tyr198Cys
**DA23872**	4504994	SNV	CDS: yjcD	Thr24Ala
**DA23872**	4601391	SNV	CDS: mutL	Lys307Arg
**DA23872**	4621196	SNV	CDS: yjfP	
**DA23872**	4642401	Deletion		
**DA23872**	4676758	SNV	RBS: RBS,	
**DA23872**	4713625	SNV	CDS: ytgA	
**DA23872**	4761505	SNV	CDS: trpS2	Tyr39Cys

**DA23872**	4767281	SNV		
**DA23874**	3060	Deletion	CDS: thrB	Asn89fs
**DA23874**	100449	SNV	CDS: kefC	Met558Val
**DA23874**	129259	SNV	CDS: leuD	Leu135Pro
**DA23874**	218255	Insertion	CDS: ligT	Ala10fs
**DA23874**	237104	Insertion	CDS: putative outer membrane protein	
**DA23874**	253245	SNV	CDS: map	
**DA23874**	272135	SNV	CDS: dnaE	Phe863Leu
**DA23874**	337446	Insertion	CDS: putative RHS-like protein	Leu120fs
**DA23874**	417817	Deletion		
**DA23874**	496416	SNV	CDS: cyoB	Asp256Asn
**DA23874**	518970	SNV	CDS: mdlB	
**DA23874**	661009	SNV	CDS: ybdB	Gln110*
**DA23874**	673098	SNV		
**DA23874**	793807	SNV	CDS: putative cytoplasmic protein	
**DA23874**	794629	SNV	CDS: nei	
**DA23874**	977144	Insertion	CDS: hypothetical protein	Gln113fs
**DA23874**	1048863	SNV	CDS: dmsC	Phe156Leu
**DA23874**	1117553	SNV	CDS: hypothetical protein	Lys232Glu
**DA23874**	1128378	Insertion	CDS: probable minor tail protein	Asn792fs
**DA23874**	1128798	SNV	CDS: probable minor tail protein	
**DA23874**	1198420	SNV		
**DA23874**	1232990	Deletion	RBS: RBS	
**DA23874**	1275289	SNV	CDS: yceD	
**DA23874**	1292479	SNV	CDS: thiK	Ile240Val
**DA23874**	1375139	Insertion	CDS: ydjA	Asp72fs
**DA23874**	1375969	Insertion	CDS: selD	Arg126fs
**DA23874**	1383031	SNV	CDS: argD	
**DA23874**	1412250	SNV	CDS: thrS	
**DA23874**	1593976	Deletion		
**DA23874**	1672348	SNV	CDS: putative outer membrane lipoprotein	Asn30Asp
**DA23874**	1689722	SNV	CDS: ydcR	Glu31Gly
**DA23874**	1696210	Deletion	CDS: ydcN	Met1?
**DA23874**	1739562	SNV	CDS: ydbH	Thr75Ala
**DA23874**	1945674	SNV	CDS: yebS	
**DA23874**	1948551	Insertion		
**DA23874**	1964193	Insertion	CDS: mig-3	Glu55fs

**DA23874**	2011348	Insertion	CDS: flhB	Arg29fs
**DA23874**	2021974	SNV	CDS: flhD	Asp31Gly
**DA23874**	2025489	SNV	CDS: otsB	
**DA23874**	2191482	Insertion	CDS: wcaH	Ala61fs
**DA23874**	2191634	SNV	CDS: wcaH, CDS: wcaG	*322Trp, Asp10Gly
**DA23874**	2237713	SNV	CDS: yegU	Phe59Ser
**DA23874**	2256471	SNV	CDS: yehV	
**DA23874**	2268330	SNV	CDS: yohF	Thr145Ala
**DA23874**	2272816	SNV	CDS: salicylate hydroxylase	Pro50Ser
**DA23874**	2337127	SNV	CDS: hypothetical protein	Ser20Gly
**DA23874**	2507250	SNV		
**DA23874**	2539831	SNV	CDS: ligA	Pro396Ser
**DA23874**	2585981	SNV	CDS: ypfG	
**DA23874**	2656147	SNV		
**DA23874**	2677887	SNV	CDS: fdx	Thr104Ala
**DA23874**	2696935	SNV	CDS: cadC	
**DA23874**	2787988	SNV	CDS: yfiF	Val156Ala
**DA23874**	2828043	Insertion	CDS: STM2689	Asp156fs
**DA23874**	2864334	SNV	CDS: terminase-like protein	Phe99Leu
**DA23874**	2923168	SNV	CDS: iroE	
**DA23874**	2949760	SNV	CDS: putative regulatory protein	
**DA23874**	2954321	SNV	CDS: nrdE	Arg612His
**DA23874**	3017731	SNV	Misc. feature: SPI-1, TTSS-1	
**DA23874**	3037974	SNV	Misc. feature: SPI-1, TTSS-1, CDS: invC	Thr3Ala
**DA23874**	3147016	SNV	CDS: RecB	Ala882Asp
**DA23874**	3160713	SNV	CDS: ptsP	Gln484*
**DA23874**	3164027	Deletion	CDS: mutH	Asn216fs
**DA23874**	3185632	SNV	CDS: putative cytoplasmic protein	
**DA23874**	3188195	SNV	CDS: stdC	Arg95Cys
**DA23874**	3206917	SNV	RBS: RBS,	
**DA23874**	3285551	SNV	CDS: putative amino acid transporter	Thr457Ala
**DA23874**	3315175	SNV	CDS: putative methyl-accepting chemotaxis protein	
**DA23874**	3326574	SNV	CDS: putative cation transporter	
**DA23874**	3376866	SNV	CDS: rpoD	Val153Ala
**DA23874**	3468761	SNV	CDS: dacB	Ala34Thr
**DA23874**	3501490	SNV	CDS: yhcG	Ala289Thr

**DA23874**	3587680	SNV	CDS: rplO	
**DA23874**	3666047	SNV	CDS: feoB	Ala634Thr
**DA23874**	3679441	SNV	CDS: malT	Ile856Val
**DA23874**	3796464	SNV	CDS: yhjL	Gln317Arg
**DA23874**	3824219	SNV	CDS: lpfC	Met801Lys
**DA23874**	3882244	SNV	CDS: putative inner membrane protein	Ala347Val
**DA23874**	3942757	SNV	CDS: yicH	Arg6Met
**DA23874**	3995090	SNV	CDS: putative regulatory protein	Val93Ala
**DA23874**	4081275	SNV	CDS: gidB	Gln169*
**DA23874**	4095010	SNV	CDS: rbsB	Ala149Val
**DA23874**	4116110	SNV	CDS: putative cytoplasmic protein	
**DA23874**	4387155	SNV		
**DA23874**	4409829	SNV	CDS: metH	
**DA23874**	4444719	SNV	CDS: yjbH	Thr670Met
**DA23874**	4532538	SNV	CDS: basR	Arg210His
**DA23874**	4647665	SNV	CDS: ytfN	Thr369Ala
**DA23874**	4671944	Deletion	Misc. feature: putative sugar kinase, ribokinase family, contains frameshift	
**DA23874**	4716168	SNV	CDS: valS	Gly828Ser
**DA23874**	4719833	SNV	CDS: pepA	Val303Ala
**DA23874**	4728461	SNV	CDS: idnK	His157Arg
**DA23874**	4739921	Insertion	CDS: putative cytoplasmic protein	Glu79fs
**DA23874**	4740249	SNV	CDS: putative cytoplasmic protein	
**DA23874**	4741935	SNV	CDS: putative ABC-type sugar/spermidine/putrescine transport system ATPase component	Gly37Arg
**DA23874**	4809426	SNV	tRNA: leuV	
**DA23874**	4809658	SNV	tRNA: leuQ	
**DA23877**	2021643	SNV	CDS: flhC	Ala24Ser
**DA23877**	3252058	SNV	CDS: metK	Gln120Leu
**DA23877**	3678860	SNV	CDS: malT	Leu662*
**DA23877**	4081563	SNV	CDS: gidB	Asp73Asn
**DA23879**	656069	MNV		
**DA23879**	1685668	Deletion	CDS: srfC	Asp536fs
**DA23879**	3677833	Deletion	CDS: malT	Tyr320fs
**DA23879**	4081582	Insertion	CDS: gidB	Gln67fs

Supplementary Table 2. Epistatic interaction terms based on background-averaged epistasis (ε) and on epistatic terms relative to the wild type sequence (λ). The different reconstructed variants are listed in binary order, and the phenotypic resistance values were linearized as log_2(MIC) to fit the linear epistasis model.

Strain number	Binary genotype[1]	Interaction terms[1]	MIC streptomycin (mg L^{-1})	log_2(MIC) *y*	Background-averaged epistasis ε	Epistatic term λ
**DA6192**	00000	*****	4	2	5.24	2
**DA40124**	00001	****1	3	1.58	0.05	-0.42
**DA39336**	00010	***1*	6	2.58	0.95	0.58
**DA40128**	00011	***11	4	2	0.11	-0.17
**DA40118**	00100	**1**	8	3	1.33	1
**DA40482**	00101	**1*1	12	3.58	0.16	1
**DA40122**	00110	**11*	12	3.58	0.05	0
**DA40381**	00111	**111	16	4	0.10	0
**DA39332**	01000	*1***	8	3	1.53	1
**DA40126**	01001	*1**1	6	2.58	0.05	0
**DA40130**	01010	*1*1*	16	4	0.45	0.42
**DA40385**	01011	*1*11	12	3.58	-0.10	0.17
**DA40120**	01100	*11**	16	4	-0.09	0
**DA40378**	01101	*11*1	16	4	-0.60	-0.58
**DA40389**	01110	*111*	32	5	1.19	0
**DA40469**	01111	*1111	32	5	1.21	0
**DA40134**	10000	1****	32	5	3.80	3
**DA40473**	10001	1***1	24	4.58	0.30	0
**DA40142**	10010	1**1*	64	6	0.41	0.42
**DA40477**	10011	1**11	96	6.58	0.40	1.17
**DA40471**	10100	1*1**	96	6.58	-0.05	0.58
**DA40483**	10101	1*1*1	96	6.58	-1.10	-0.58
**DA40383**	10110	1*11*	128	7	0.10	-0.58
**DA40463**	10111	1*111	128	7	0.21	-1
**DA40138**	11000	11***	96	6.58	0.84	0.58
**DA40475**	11001	11**1	192	7.58	0.52	1.42
**DA40387**	11010	11*1*	192	7.58	-0.10	-0.42
**DA40465**	11011	11*11	256	8	-0.55	-1.75
**DA40379**	11100	111**	256	8	0.40	-0.17
**DA40461**	11101	111*1	192	7.58	-0.04	-1.25
**DA40467**	11110	1111*	768	9.58	2.38	1.17
**DA40479**	11111	11111	1024	10	2.42	2.42

[1] The five genes are *gidB, nuoG, cyoB, znuA* and *trkH*, corresponding to the bits in the binary genotype column in left-to-right order.

Supplementary Table 3. Relative growth rate and mutation rates of isolated mutants. The growth rate of the ancestral wild type strain DA6192 is set to 1.0. Fitness was measured as exponential growth rate in Mueller Hinton medium at 37°C. Mutation rates μ are the estimated rates per cell per division corrected by the plating efficiency, and they were calculated using the bz-rates web tool[1].

Strain	Relative growth rate	Relative growth rate SD	Mutation rate *μ*
**DA6192**	1	0.0262	2.1e-09
**DA23868**	0.96	0.0177	1.9e-07
**DA23869**	0.89	0.0260	2.9e-07
**DA23872**	0.92	0.0177	3.2e-07
**DA23874**	0.98	0.0168	3.9e-07

Supplementary Table 4. Populations evolved above MIC. Mutations in the 5 populations of *S. typhimurium* evolved in MH medium containing 200 mg L^{-1} streptomycin for 100 generations compared to wild type. Mutation types are single-nucleotide variants (SNVs), deletions and insertions, and "fs" represents a frame shift mutation. All mutations detected in >3 reads are listed.

Strain	Position	Type	Frequency	Annotation	Amino acid change
**DA60384**	3602590	SNV	100.00	CDS: rpsL	Lys43Arg
**DA60384**	2174308	Deletion	23.81	CDS: rfbI	Gly268fs
**DA60385**	3602590	SNV	100.00	CDS: rpsL	Lys43Arg
**DA60385**	2173959	SNV	36.36	CDS: rfbF	Tyr55Asp
**DA60386**	3602590	SNV	100.00	CDS: rpsL	Lys43Arg
**DA60386**	2171585	SNV	20.41	CDS: rfbH	Pro218Leu
**DA60386**	1853325	SNV	11.43		
**DA60386**	2173649	SNV	10.00	CDS: rfbF	Pro158Leu
**DA60387**	3602590	SNV	100.00	CDS: rpsL	Lys43Arg
**DA60387**	2168404	SNV	36.36	CDS: rfbV	Pro72Leu
**DA60387**	2852125	SNV	35.90	CDS: hypothetical protein	
**DA60387**	2852155	SNV	14.29	CDS: hypothetical protein	
**DA60387**	2854636	SNV	11.11	CDS: phage tail-like protein	
**DA60388**	3602590	SNV	100.00	CDS: rpsL	Lys43Arg
**DA60388**	2168088	Deletion	31.58	CDS: rfbV	Glu178fs
**DA60388**	3052876	SNV	14.29		

Supplementary Table 5. Isolated resistant mutants. Mutants of *S. typhimurium* clones isolated from serial passage in MH medium containing 1 mg L^{-1} streptomycin for 900 generations.

Strain	Genotype	Reference
**DA23866**	Mutant isolated by plating a serial passage population on 256 mg/L streptomycin, Lineage 10, Clone 1	This study
**DA23867**	Mutant isolated by plating a serial passage population on 256 mg/L streptomycin, Lineage 10, Clone 2	This study
**DA23868**	Mutant isolated by plating a serial passage population on 256 mg/L streptomycin, Lineage 10, Clone 3	This study
**DA23869**	Mutant isolated by plating a serial passage population on 256 mg/L streptomycin, Lineage 19, Clone 1	This study
**DA23870**	Mutant isolated by plating a serial passage population on 256 mg/L streptomycin, Lineage 19, Clone 2	This study
**DA23871**	Mutant isolated by plating a serial passage population on 256 mg/L streptomycin, Lineage 19, Clone 3	This study
**DA23872**	Mutant isolated by plating a serial passage population on 192 mg/L streptomycin, Lineage 16, Clone 1	This study
**DA23873**	Mutant isolated by plating a serial passage population on 192 mg/L streptomycin, Lineage 16, Clone 2	This study
**DA23874**	Mutant isolated by plating a serial passage population on 128 mg/L streptomycin, Lineage 7, Clone 1	This study
**DA23875**	Mutant isolated by plating a serial passage population on 128 mg/L streptomycin, Lineage 7, Clone 2	This study
**DA23876**	Mutant isolated by plating a serial passage population on 128 mg/L streptomycin, Lineage 7, Clone 3	This study
**DA23877**	Mutant isolated by plating a serial passage population on 96 mg/L streptomycin, Lineage 11, Clone 1	This study
**DA23878**	Mutant isolated by plating a serial passage population on 96 mg/L streptomycin, Lineage 11, Clone 2	This study
**DA23879**	Mutant isolated by plating a serial passage population on 96 mg/L streptomycin, Lineage 9, Clone 1	This study
**DA23880**	Mutant isolated by plating a serial passage population on 96 mg/L streptomycin, Lineage 9, Clone 2	This study
**DA23881**	Mutant isolated by plating a serial passage population on 96 mg/L streptomycin, Lineage 9, Clone 3	This study

Supplementary Table 6. Primers used for strain reconstruction. Primers with a name containing "dup" were used to amplify and insert a *cat-sacB* cassette near each gene using Lambda-Red recombineering, generating a marker-held tandem duplication, while primers with a name containing "scr" were used to screen and sequence the gene for mutations. Primer names containing 'csb' were used to amplify a *cat-sacB* cassette that was inserted into the target genes and subsequently replaced by single stranded oligos (ssLR) resulting in a gene knockout or by a *dksA* variant from *Pseudomonas aeruginosa* (*dksA2*).

Name	Sequence
**gidB_dup_F**	ATGCCCGATGCGCTTTCGCTTATCGGGCCTACAAATTTGTAGGCTGGAGCTGCTTC
**gidB_dup_R**	AAACGACGCGGCTTGTGTTAAAAATCGACAGGTATAACGCATATGAATATCCTCCTTAGTTCC
**gidB_scr_F**	CGATTCGATTTTTAGCAGAC
**gidB_scr_R**	GAAAAAACAAGGTATGCTGC
**trkH_dup_F**	AATACCATCATCGCTATTTTCCTGCTGATCTCCGGTTGTAGGCTGGAGCTGCTTC
**trkH_dup_R**	CCAGTAGTCCAACGATTCGGGTAATGGCGCGAAAATGCATATGAATATCCTCCTTAGTTCC
**trkH_scr_F**	CCGAATCGTTGGACTACT
**trkH_scr_R**	CGGAACATAGCAGCAAGAC
**cyoB_dup_F**	CGATGAGATTACTAAGGCAGGGCTGAAAAATGGCAACTGATGTAGGCTGGAGCTGCTTC
**cyoB_dup_R**	TAATTTTCCGAACATCTTATCTTCCTCAACCCTTGGAGTTTTATATCATAAATATCTGTGTCATATGAATATCCTCCTTAGTTCC
**cyoB_scr_F**	TTACCCCAGGTTTCGTTCA
**cyoB_scr_R**	GGTCTGATGAACCTGGTG
**nuoG_dup_F**	TGGCGCGCGTCTTGAGGATCTGCGGGAGGCGCAACAATGACTTGTAGGCTGGAGCTGCTTC
**nuoG_dup_R**	ATCGTAGCCATTAGCATGCTTCCAAATTAAAAATCGTTAATTATATCATAAATATCTGTGTCATATGAATATCCTCCTTAGTTCC
**nuoG_scr_F**	CCTCACCCAAGACCAGTA
**nuoG_scr_R**	ATACCAAAACGCGGAAGA
**znuA_dup_F**	GGGAAATGGTCACTCATGATTGAATTATTACTGCCCGGCTGTAGGCTGGAGCTGCTTC
**znuA_dup_R**	GGACTCACCCCGCTCGGTCACTTTACCGTGAACCCTGAGACATATGAATATCCTCCTTAGTTCC
**znuA _scr_F**	GTGCCTCAAAATCCTTCAG
**znuA _scr_R**	CGGAGAGTTTTTGCATGG
**znuA_csb_P1**	CGTCGGCAGCCTGGGTTGCACTCCCCCAAAGCGCGGCGGATGTAGGCTGGAGCTGCTTC
**znuA_csb_P2**	CATTATGTTACAGAAAAATACGCTTCTTTTCGCAGCATTACATATGAATATCCTCCTTAGTTCC
**znuA_del_ssLR**	TATCACATTTCACACATTCATTACGATGATTAGTCGCATTCGAGGAAGTGAATACGTGCAACAGATAGCCCGCTCTGTCG
**screen_znuA_fw**	TTATCAGAAGCGGCAAGT
**screen_znuA_rv**	ACCAAATGAGACCGAGAC
**dksA_csb_P1**	AAACTCCCGCCTGTCATAAATAGGGTAGAAACGAACGGGAGTGTAGGCTGGAGCTGCTTC
**dksA_csb_P2**	CGAACATGGGGATCGATAGTGCGTGTTAAGGAGAAGCAACCATATGAATATCCTCCTTAGTTCC
**dksA_del_ssLR**	CGAACATGGGGATCGATAGTGCGTGTTAAGGAGAAGCAACTCCCGTTCGTTTCTACCCTATTTATGACAGGCGGGAGTTT
**PA_dksA2_LT2-rpl_fw**	AAACTCCCGCCTGTCATAAATAGGGTAGAAACGAACGGGATCAGTTGTGCCGCACGTG
**PA_dksA2_LT2-rpl_rv**	CGAACATGGGGATCGATAGTGCGTGTTAAGGAGAAGCAACATGACCGAACAGGAACTGC

Supplementary Table 7. Unintended mutations in the reconstructed strains. The mutations in *yebB* and *lon* were co-transduced from the isolated mutants, while the mutations in *hemG* and *gyrA* appeared during the reconstruction process.

Strain	*yebB*	*hemG*	*lon*	*gyrA*
**DA40124**	wt	P81L	wt	wt
**DA40120**	wt	wt	P12syn	wt
**DA40130**	S54G	wt	wt	wt
**DA40122**	wt	wt	P12syn	wt
**DA40473**	wt	P81L	wt	A386T
**DA40126**	wt	P81L	wt	wt
**DA40482**	wt	P81L	P12syn	wt
**DA40128**	wt	P81L	wt	wt
**DA40387**	S54G	wt	wt	wt
**DA40383**	S54G	wt	P12syn	wt
**DA40389**	S54G	wt	wt	wt
**DA40475**	wt	P81L	wt	wt
**DA40483**	wt	P81L	wt	wt
**DA40378**	wt	P81L	wt	wt
**DA40477**	S54G	P81L	wt	wt
**DA40385**	S54G	P81L	wt	wt
**DA40381**	S54G	P81L	P12syn	wt
**DA40467**	S54G	wt	wt	wt
**DA40461**	wt	P81L	wt	wt
**DA40465**	S54G	P81L	wt	wt
**DA40463**	S54G	P81L	P12syn	wt
**DA40469**	S54G	P81L	wt	wt
**DA40479**	S54G	P81L	wt	wt

Supplementary Table 8. Primers used for RT-PCR

Name	Sequence
**qPCR_aadA_fw**	GGA GGT CAC TGT CGT GCT TT
**qPCR_aadA_rv**	ACC ATG TCC CAG TCC TGC T
**qPCR_iraP_fw**	GGG CAC TTG AAG GCG TAA
**qPCR_iraP_rv**	GCG CAG CAA TTC CGT ATC
**qPCR_cysG_fw**	CCA CAG GAG GAA ATC AAC CA
**qPCR_cysG_rv**	CGT ACC ACG CGT TTA CCT TT
**qPCR_hcaT_fw**	ACG ATT ACC AGG CGA TTC TG
**qPCR_hcaT_rv**	CGC TGG GAC GTA ACA ACA TAC

Supplementary Reference

1. Gillet-Markowska, A., Louvel, G. & Fischer, G. bz-rates: A Web Tool to Estimate Mutation Rates from Fluctuation Analysis. G3 (*Bethesda*) **5**, 2323-2327 (2015).
